# The Early Effects of Rapid Androgen Deprivation on Human Prostate Cancer

**DOI:** 10.1016/j.eururo.2015.10.042

**Published:** 2016-08

**Authors:** Greg L. Shaw, Hayley Whitaker, Marie Corcoran, Mark J. Dunning, Hayley Luxton, Jonathan Kay, Charlie E. Massie, Jodi L. Miller, Alastair D. Lamb, Helen Ross-Adams, Roslin Russell, Adam W. Nelson, Matthew D. Eldridge, Andrew G. Lynch, Antonio Ramos-Montoya, Ian G. Mills, Angela E. Taylor, Wiebke Arlt, Nimish Shah, Anne Y. Warren, David E. Neal

**Affiliations:** a Cancer Research UK Cambridge Institute, Li Ka Shing Centre, Cambridge, UK; b Department of Urology, Cambridge University Hospitals NHS Trust, Cambridge, UK; c Department of Pathology, Cambridge University Hospitals NHS Trust, Cambridge, UK; d Prostate Cancer Research Group, Nordic EMBL Partnership, Centre for Molecular Medicine Norway (NCMM), University of Oslo, Oslo, Norway; e Departments of Cancer Prevention and Urology, Institute of Cancer Research and Oslo University Hospitals, Oslo, Norway; f Prostate Cancer UK/Movember Centre of Excellence for Prostate Cancer Research, Centre for Cancer Research and Cell Biology, Queens University Belfast, Belfast, UK; g Centre for Endocrinology, Diabetes and Metabolism, University of Birmingham, Egbaston, Birmingham, UK; h Nuffield Department of Surgery, University of Oxford, John Radcliffe Hospital, Headington, Oxford, UK; i University College Hospitals NHS Trust, UK; j University College London, London, UK

**Keywords:** Prostate cancer, Androgen receptor, Castration, Clinical trial, Immunohistochemistry, Gene transcription

## Abstract

The androgen receptor (AR) is the dominant growth factor in prostate cancer (PCa). Therefore, understanding how ARs regulate the human transcriptome is of paramount importance. The early effects of castration on human PCa have not previously been studied 27 patients medically castrated with degarelix 7 d before radical prostatectomy. We used mass spectrometry, immunohistochemistry, and gene expression array (validated by reverse transcription-polymerase chain reaction) to compare resected tumour with matched, controlled, untreated PCa tissue. All patients had levels of serum androgen, with reduced levels of intraprostatic androgen at prostatectomy. We observed differential expression of known androgen-regulated genes (*TMPRSS2, KLK3, CAMKK2, FKBP5*). We identified 749 genes downregulated and 908 genes upregulated following castration. AR regulation of α-methylacyl-CoA racemase expression and three other genes (*FAM129A, RAB27A*, and *KIAA0101*) was confirmed. Upregulation of oestrogen receptor 1 (ESR1) expression was observed in malignant epithelia and was associated with differential expression of ESR1-regulated genes and correlated with proliferation (Ki-67 expression).

**Patient summary:**

This first-in-man study defines the rapid gene expression changes taking place in prostate cancer (PCa) following castration. Expression levels of the genes that the androgen receptor regulates are predictive of treatment outcome. Upregulation of oestrogen receptor 1 is a mechanism by which PCa cells may survive despite castration.

## Take Home Message

This first-in-man study defines the rapid gene expression changes taking place in prostate cancer (PCa) following castration. Upregulation of ESR1 is a mechanism by which PCa cells may survive despite castration.

Prostate cancer (PCa) is the second most common cause of cancer death in men in the developed world [1]. The androgen receptor (AR) controls PCa growth. Previously, studies of the long-term effects (>3 mo) of medical castration have demonstrated differing transcriptional response following luteinising hormone-releasing hormone (LHRH) analogue and antiandrogens [2] and have implicated Wnt/β-catenin signalling in castration-resistant PCa [3]. We used a new drug called degarelix to rapidly decrease testosterone levels and inform the early in vivo response of human PCa to castration.

## 1 Patients and methods

### 1.1 Study approval

We obtained full ethics approval for all elements of studies NCT01852864 (REC ref:11/H0311/2) and NCT00967889 (REC ref:01/4/061).

### 1.2 Clinical sample collection

Twenty-seven patients with high-risk PCa (according to the D’Amico classification) [4] were administered 240 mg subcutaneous degarelix (donated by Ferring Pharmaceuticals Inc, Parsippany, NJ, USA) 7 d before radical prostatectomy (RP) (Supplementary Fig. 1a). These patients were matched for risk factors (age, serum prostate-specific antigen, tumour grade and stage) with 20 patients undergoing RP without neoadjuvant degarelix (Supplementary Table 1). Study and control patients underwent surgery contemporaneously using standard operative technique, with similar ischemic time before sampling as described [5]. Serum samples were obtained at 8 am prior to surgery.

### 1.3 Assay of androgen levels in body fluids and tissue

Fresh frozen prostate cores were homogenised, and steroids were extracted and quantified by liquid chromatography coupled with tandem mass spectrometry.

### 1.4 Gene expression profiling

We hybridised mRNA onto Illumina HumanHT-12 v4 Expression BeadChips (Illumina, San Diego, CA, USA).

### 1.5 Accession numbers

Study data are deposited in the National Center for Biotechnology Information Gene Expression Omnibus under accession number GSE72920. Expression data were validated for known AR-regulated genes (*KLK3, FASN*), genes found to be AR regulated in our expression data with known AR binding sites in promoter regions [6] (*FAM129A, KIAA0101, RAB27A*) and genes of biological importance (*AMACR, ESR1*, cyclin D1) by reverse transcription-polymerase chain reaction (RT-PCR) and immunohistochemistry (IHC).

### 1.6 Quantitative real-time polymerase chain reaction

We performed RT-PCR in triplicate using SYBR Green and the 7900HT Real-Time PCR System (Applied Biosystems, Foster City, CA, USA).

### 1.7 Human tissue microarray construction and immunohistochemistry

PCa samples (index lesion) from 27 patients were identified on the haematoxylin and eosin–stained sections from formalin-fixed paraffin–embedded RP specimens and marked (A.W.). Triplicate 2-mm tumour cores were taken from these areas and re-embedded in human tissue microarray (TMA) paraffin blocks.

We used a BOND-III automated stainer (Leica Biosystems, Buffalo Grove, IL, USA) and Bond Polymer Refine Detection Kit (Leica Biosystems) for IHC. Scoring was by two blinded observers (including A.W.) until consensus was reached. Staining intensity was classified as 0 (none), 1 (weak), 2 (moderate), and 3 (high). For nuclear staining (*ESR1*, Ki-67, and *CCND1*), we used the following modified scoring systems to quantify the number of moderately or intensely staining nuclei as a proportion of all the malignant epithelial cells: *ESR1* and *CCND1* staining (0, no intensely staining positive nuclei; 1, 0.1–10% intensely stained nuclei per core; 2, 10–20% intensely staining nuclei per core; 3, >20% stained nuclei per core) and Ki-67 staining (0, no positive nuclei; 1, 1–5% positive nuclei; 2, 5–10% positive nuclei; 3, >10% positive nuclei).

### 1.8 Statistical analysis

All data are quoted as median plus or minus interquartile range, and *p* values <0.05 were considered significant. We use the paired *t* test for paired data; for unpaired data, we used an unpaired *t* test for parametric data and the Mann-Whitney test for nonparametric data. For TMA IHC scoring analysis, we computed the mean intensities for each sample and performed a Mann-Whitney test.

### 1.9 Gene set enrichment analysis

We used Gene Set Enrichment Analysis (GSEA) version 2.0.14 (The Broad Institute, Cambridge, MA, USA). We used the Reactome version 3.0 database to analyse differentially expressed genes (DEGs) following castration for enrichment of predefined gene sets. Using the JASPAR database in the Pscan Web interface (version 1.3), we analysed 500 bases upstream of the top 500 DEGs for enriched transcription factor binding sites (Fig. 2C).

## 2 Results

**Fig. 1:**
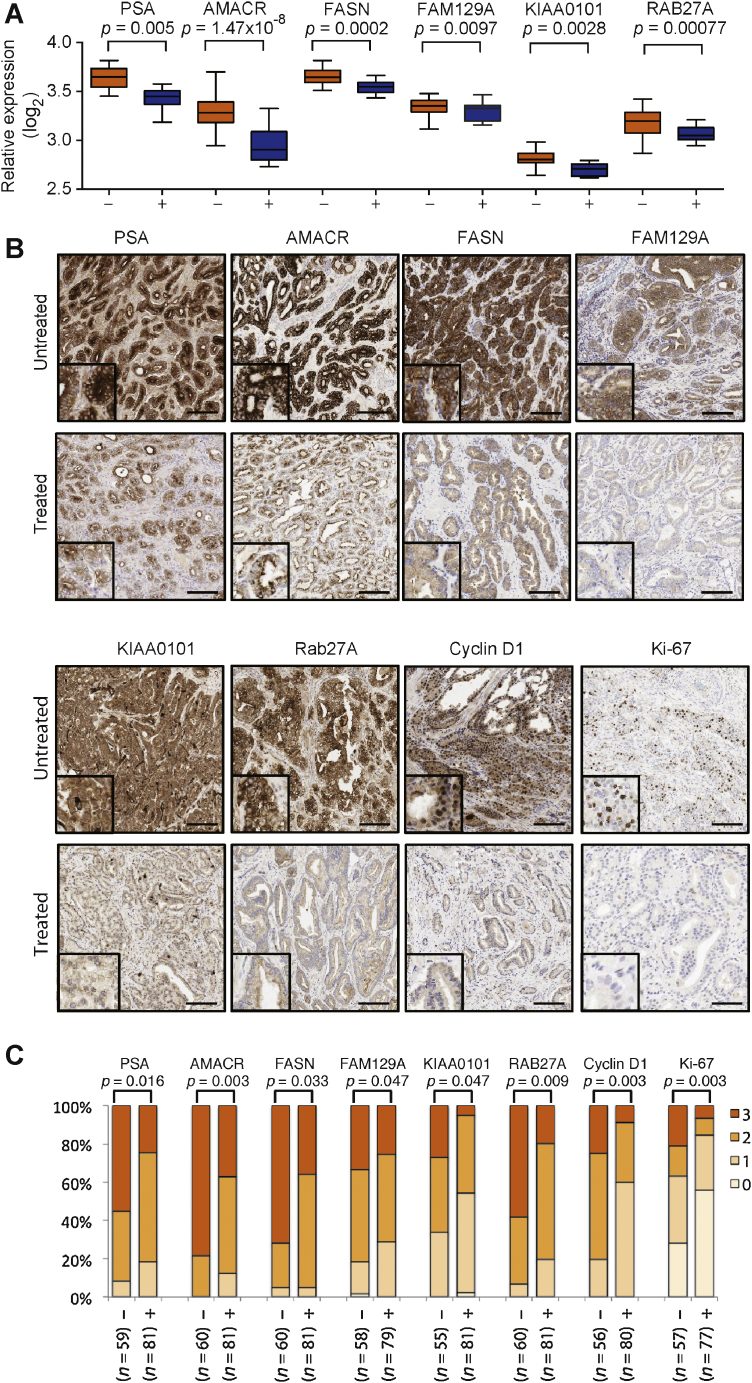
Degarelix treatment regulates expression of known androgen receptor (AR)–regulated genes and genes not previously identified as AR regulated. (A) Messenger RNA expression levels of known AR-regulated genes *PSA* and *FASN* as well as putative AR-regulated genes *AMACR, FAM129A, RAB27A*, and *KIAA0101* were decreased in degarelix-treated (+) prostate cancer (PCa) samples compared with untreated (−) samples. (B) Representative immunohistochemistry (IHC) images of PCa samples from degarelix-treated and untreated patients. Inlay = ×2 magnification. Scale bars = 250 μm. (C) Bar chart to show the distribution of staining intensity of a tissue microarray made up of PCa samples from untreated (−) and degarelix-treated (+) patients stained by IHC (0 [no staining] to 3 [intense staining]; samples in triplicate; sample sizes [*n*] evaluated in each analysis include the number of subjects [20 or 27] times 3 minus missing or damaged samples; staining intensity 0 [none] to 3 [strong]). Mean intensities from replicate samples for each patient were used to calculate statistical significance when comparing treated and untreated groups (Mann-Whitney test).

We saw no differences in the tumour or patient characteristics of the study and control cohorts (Supplementary Table 1). We saw a rapid decrease in the serum testosterone at 7 d after degarelix administration in all treated patients (11.73 ± 5.08 vs 1.19 ± 0.63 nmol/l) (Supplementary Fig. 1B), paralleled by a decrease in intratumoural androgens (Supplementary Fig. 1C). We identified 749 genes downregulated and 908 genes upregulated in response to castration. Expression levels of known AR-regulated genes—including *TMPRSS2, FKBP5, KLK3*, and *FASN*—were among those most strongly affected by treatment (Supplementary Table 2 and 3). We validated differential expression for eight genes by RT-PCR and IHC (Fig. 1B and 1C).

Degarelix treatment decreased nuclear expression of proliferation marker Ki-67 protein and cell cycle progression (expression of nuclear *CCND1*) (Fig. 1B and 1C). We observed and validated decreased expression of three genes with limited evidence of AR regulation but with AR binding sites in their promoter regions (*RAB27A, KIAA0101*, and *FAM129A*) following degarelix treatment (Fig. 1A–1C)

### 2.1 ESR1 expression is upregulated in malignant epithelia, is pro-proliferative, and is associated with transcription of known ESR1 target genes

**Fig. 2:**
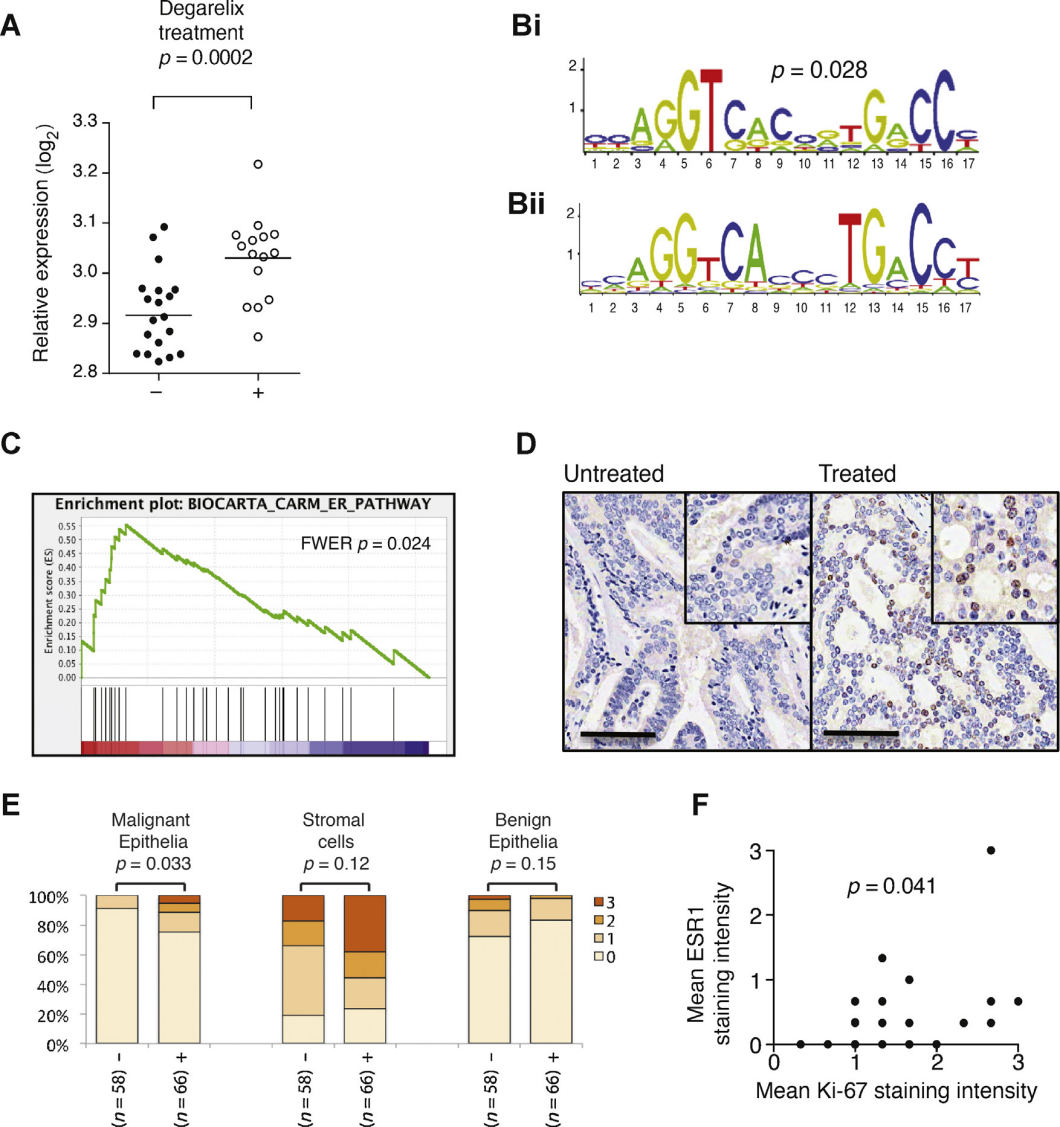
*ESR1* is upregulated in response to degarelix treatment. (A) Dot plots to show mRNA expression levels of *ESR1* in untreated and degarelix-treated prostate cancer (PCa) samples by expression array. (Bi) Using the Gene Set Enrichment Analysis (GSEA) to examine the distribution of known genes with the *ESR1* binding motif, shown within their promoter regions, we found that these genes were enriched among genes that degarelix treatment upregulated when they were ranked by their statistical significance. The *ESR1* binding motif analysed was described previously [7]. (Bii) This *ESR1* binding motif closely matches the validated, experimentally derived *ESR1* binding motif [10] shown here. (C) GSEA demonstrates enrichment of factors known to be involved with *ESR1* signalling (from the National Cancer Institute BIOCARTA curated database) among genes differentially expressed in response to degarelix ranked by statistical significance. For this analysis, the degree to which the genes were enriched is defined by the running sum statistic called the *normalised enrichment score*, which was 2.036 (false discovery rate *q*-value of 0.027; *p* = 0.024). (D) Representative images of PCa samples stained by immunohistochemistry (IHC) for *ESR1* in untreated and degarelix-treated patients with intense nuclear staining seen in the malignant epithelia of the treated but not the untreated samples. Scale bars = 250 μm. (E) IHC *ESR1* staining was increased in treated (+) compared with untreated (−) PCa samples in malignant epithelia but not cancer-associated stroma or benign epithelia (samples in triplicate; sample sizes [*n*] indicate the number of subjects [20 or 27] times 3 minus missing or damaged samples on the human tissue microarray; staining intensity 0 [none] to 3 [strong]). Mean intensities from replicate samples for each patient were used to calculate statistical significance when comparing treated and untreated groups (Mann-Whitney test). (F) Graph shows correlation between intensity of staining by IHC for *ESR1* and Ki-67. Spearman's ρ correlation coefficient of 0.338 (*p* = 0.041).

Degarelix treatment upregulated *ESR1* mRNA (Fig. 2A). Using GSEA, we demonstrated enrichment of genes that had an *ESR1* binding motif (Fig. 2B) [7] within the promoter region and genes known to be involved in *ESR1* signalling (Fig. 2C) among those genes whose expression was upregulated with degarelix treatment (Fig. 2C).

IHC demonstrated expression of *ESR1* by stromal cells but not epithelial cells (benign or malignant) in untreated samples, but 24% of degarelix-treated cancers stained positive for *ESR1* in malignant epithelia compared with 8% of untreated samples (Fig. 2E). Despite an overall decrease in the expression of the proliferation marker Ki-67 in malignant glands following degarelix treatment (Fig. 1B and 1C); in those glands with increased nuclear *ESR1* staining, proliferation was upregulated (Fig. 2F).

## 3 Discussion

In addition to identifying a large number of AR-regulated genes, we have confirmed AR regulation of three genes (*RAB27A, FAM129*, and *KIAA0101*) not proven to be AR regulated in vivo as well as *AMACR*, for which data were conflicting. *ESR1* is essential for prostate carcinogenesis and implicated in PCa growth control [8]. Polymorphism of *ESR1* is associated with PCa prognosis [9].

We observed rapid upregulation of *ESR1* expression with castration (Fig. 2A). Increased *ESR1* mRNA expression following prolonged castration using LHRH analogues has been observed [2], [3]. Upregulation of *ESR1* expression may represent an intrinsic mechanism by which some malignant prostate epithelial cells proliferate despite castration.

  ***Author contributions:*** Greg L. Shaw had full access to all the data in the study and takes responsibility for the integrity of the data and the accuracy of the data analysis.  

*Study concept and design:* Shaw, Whitaker, Corcoran, Mills, Neal, Massie, Lynch, Nelson.

*Acquisition of data:* Shaw, Corcoran, Luxton, Kay, Miller, Warren, Arlt, Taylor, Shah, Ross-Adams.

*Analysis and interpretation of data:* Shaw, Dunning, Lamb, Eldridge, Russell, Ramos-Montoya.

*Drafting of the manuscript:* Shaw, Whitaker, Lamb, Nelson, Russell.

*Critical revision of the manuscript for important intellectual content:* Shaw, Mills, Neal, Massie.

*Statistical analysis:* None.

*Obtaining funding:* Shaw, Neal.

*Administrative, technical, or material support:* None.

*Supervision:* Mills, Neal.

*Other* (specify): None.  

***Financial disclosures:*** Greg L. Shaw certifies that all conflicts of interest, including specific financial interests and relationships and affiliations relevant to the subject matter or materials discussed in the manuscript (eg, employment/affiliation, grants or funding, consultancies, honoraria, stock ownership or options, expert testimony, royalties, or patents filed, received, or pending), are the following: None.  

***Funding/Support and role of the sponsor:*** The Academy of Medical Sciences, the National Institute for Health Research, Cancer Research UK, and National Cancer Research provided funding for the design and conduct of the study as well as the collection, analysis, and interpretation of data.  

***Acknowledgments:*** The authors thank CRUK; the NIHR; the Academy of Medical Sciences (RG:63397); the National Cancer Research Prostate Cancer: Mechanisms of Progression and Treatment (ProMPT) collaborative (G0500966/75466); Hutchison Whampoa Limited; the Human Research Tissue Bank (Addenbrooke's Hospital, supported by the NIHR Cambridge BRC); and Cancer Research UK. We would like to thank those men with prostate cancer and the subjects who have donated their time and their samples to the Cambridge Biorepository that were used in this research. We also acknowledge the support of the research staff in S4, who so carefully curated the samples and the follow-up data (Jo Burge, Marie Corcoran, Anne George, and Sara Stearn).
