# Supplementary material for: The Early Effects of Rapid Androgen Deprivation on Human Prostate Cancer
Source: Eur Urol. 2016 Aug;70(2):214–8. doi: 10.1016/j.eururo.2015.10.042 (PMC4926724; doi:10.1016/j.eururo.2015.10.042)
Supplement: Supplementary file 1 [file mmc1.doc]

**Supplement 1 – Supplementary Methods**

**Steroid hormone quantification**

Fresh frozen prostate cores were homogenised using a Precellys 24 homogenizer (Bertin Technologies, Montigny-le-Bretonneux, France) and suspended in 500 µl of distilled water. Serum samples for steroid assays were taken at 8 am. Steroids were extracted from plasma and prostate biopsies by liquid/liquid extraction using methyl tertiary butyl ether. Steroid levels were quantified by liquid chromatography tandem mass spectrometry using a Waters Xevo mass spectrometer (Waters, Milford, MA, USA) and ACQUITY UPLC chromatography system (Waters), with an HSS T3 1.8-µm, 1.2-mm x 50-mm column. Steroids were eluted using a methanol/water mixture with 0.1% formic acid and quantified after calibration on a series of standards ranging from 0.5 ng/ml to 500 ng/ml.

**mRNA isolation**

We isolated mRNA (AllPrep DNA/RNA Mini Kit, Qiagen, Venlo, Netherlands), quantified it (NanoDrop, Thermo Scientific, Waltham, MA, USA), and applied quality controls (2100 Bioanalyzer, Agilent Technologies, Santa Clara, CA, USA). Fifteen of 20 human processed cores from the treated patients and 20 of 20 of the controls generated mRNA of adequate quality (RNA integrity number score >8) for hybridization in a randomised distribution onto Illumina HumanHT-12 v4 Expression BeadChips (Illumina, San Diego, CA, USA).

**Quantitative real-time polymerase chain reaction**

We performed real-time polymerase chain reaction (RT-PCR) in triplicate in 10-µl reactions containing 2 pmol primers (Sigma-Aldrich, St. Louis, MO, USA), 5 µl of SYBR Green, and 10 ng of cDNA template using the Applied Biosystems 7900HT Real-Time PCR System (Applied Biosystems, Foster City, CA, USA).

Primer sequences used for RT-PCR:

ESR1-f-TGATTGGTCTCGTCTGGCG,r-CATGCCCTCTACACATTTTCCC

KLK3-f-AGAAGCATTCCCAACCCTG,r-GTCGTGGCTGGAGTCATC

AMACR-f-TTCTGCAGCGGGAAAATCCA,r- GAGAGAACACCTGACAAAGCC

FASN-f-AAGGACCTGTCTAGGTTTGATGC,r- TGGCTTCATAGGTGACTTCCA

Cyclin D1-f- CTGAGGAGCCCCAACAACTT,r- CAGTCCGGGTCACACTTGAT

FAM129A-f-CCTTTCTTTGCTCAATGCAGGGCT,r-AGGGCACTGATGTTCATCCATCCA RAB27A-f-TCCCCGCTATATTCCCATTGC,r- CTCGGCATAAGCACCAGCTA

KIAA0101-f-TGTCCAGTGAAACACCCTCG,r- CACTCGATCGCCTCACCTTT

RPLP2-f- AAGAAGATCTTGGACAGCGTGGGT,r-TACCCTGGGCAATGACGTCTTCAA.

We calculated cycle threshold (Ct) values for all conditions and normalised the expression of target genes against the expression of *RPLP2* endogenous control gene using the ΔΔCt method. We validated differential expression on expression array for *ESR1, KLK3, AMACR, FASN*, cyclin D1, *FAM129A, KIAA0101*, and *RAB27A*.

**Data analysis**

We processed data from the HumanHT-12 arrays by using the beadarray Bioconductor package [1]. We summarised data on the log2 scale, and then quantile-normalised as described. We used the limma Bioconductor package to carry out differential expression analysis [2], with a 5% false discovery rate.

We defined the promoter region as 500 bases upstream of the transcription start site for gene set enrichment analysis (GSEA) analysis. We performed GSEA according to the described methodology [3].

For tissue microarray analysis, we calculated the *p* values with reference to 10 000 test statistics generated. We repeated this procedure for each biomarker and subsequently adjusted the *p* values for multiple testing using the Benjamini-Hochberg method [4].

**Supplementary references**

[1] Dunning MJ, Smith ML, Ritchie ME, Tavaré S. beadarray: R classes and methods for Illumina bead-based data. Bioinformatics 2007;23:2183–4.

[2] Smyth GK. Limma: linear models for microarray data. In: In Gentleman R, Carey V, Huber W, Irizarry R, Dudoit S, editors. Bioinformatics and Computational Biology Solutions in R and Bioconductor. New York, NY: Springer-Verlag; 2005. p. 397–420.

[3] Subramanian A, Tamayo P, Mootha VK, et al. Gene set enrichment analysis: a knowledge-based approach for interpreting genome-wide expression profiles. Proc Natl Acad Sci U S A 2005;102:15545–50.

[4] Benjamini Y, Hochberg Y. Controlling the false discovery rate: a practical and powerful approach to multiple testing. J Royal Stat Soc B 1995;57:289–300.

[5] Massie CE, Lynch A, Ramos-Montoya A, et al. The androgen receptor fuels prostate cancer by regulating central metabolism and biosynthesis. EMBO J 2011;30:2719–33.

[6] Sharma NL, Massie CE, Ramos-Montoya A, et al. The androgen receptor induces a distinct transcriptional program in castration-resistant prostate cancer in man. Cancer Cell 2013;23:35–47.

[7] DePrimo SE, Diehn M, Nelson JB, et al. Transcriptional programs activated by exposure of human prostate cancer cells to androgen. Genome Biol 2002;3:RESEARCH0032.
