# Supplementary material for: The Early Effects of Rapid Androgen Deprivation on Human Prostate Cancer
Source: Eur Urol. 2016 Aug;70(2):214–8. doi: 10.1016/j.eururo.2015.10.042 (PMC4926724; doi:10.1016/j.eururo.2015.10.042)
Supplement: Supplementary file 2 [file mmc2.doc]

**Supplementary Table 1 – Baseline characteristics of degarelix-treated and untreated cohorts**

|  | Degarelix treated (*n* = 20), mRNA, group A | Degarelix treated included on array (*n* = 15), mRNA, group A− | Degarelix treated (*n* = 27), IHC, group B | Untreated control (*n* = 20), group C | *p* value *n* = 20 cohort vs controls, A vs B | *p* value *n* = 27 cohort vs controls, A vs C | *p* value *n* = 27 cohort vs controls A− vs C |
| --- | --- | --- | --- | --- | --- | --- | --- |
| Age, yr, median (range) | 64 (47–69) | 63 (47–69) | 64(47–69) | 61.5 (51–70) | 0.29* | 0.28* | 0.35* |
| PSA, ng/ml, median (range) | 9.2 (5.5–23) | 8.9 (5.5–23) | 9.1 (5.4–32) | 9 (5.2–13.89) | 0.73* | 0.95* | 0.97* |
| Clinical stage |  |  |  |  |  |  |  |
| cT1 | 1 (5%) | 1 (6.7%) | 1 (3.7%) | 3 (15%) | 0.73** | 0.52** | 0.34** |
| cT2 | 14 (70%) | 13 (73.3%) | 21 (77.8%) | 13 (65%) |
| cT3 | 5 (25%) | 3 (20%) | 5 (14.4%) | 4 (20%) |
| Biopsy Gleason sum |  |  |  |  |  |  |  |
| 6 | 1 (5%) | 1 (6.7%) | 1 (3.7%) | 2 (10%) | 0.38** | 0.19** | 0.07** |
| 7 | 14 (70%) | 12 (80%) | 17 (62.9%) | 15 (75%) |
| 8 | 4 (20%) | 2 (13.3%) | 6 (22.2%) | 2 (10%) |
| 9 | 0 | 0 | 1 (3.7%) | 1 (5%) |
| 10 | 1 (5%) | 0 | 1 (3.7%) | 0 |
| Prostatectomy Gleason sum† |  |  |  |  |  |  |  |
| 7 | 17 (85%) | 14 (93.3%) | 24 (88.8%) | 16 (80%) | 0.80** | 0.87** | 0.87** |
| 8 | 0 | 0 | 0 | 3 (15%) |
| 9 | 3 (15%) | 1 (6.7%) | 3 (11.1%) | 1 (5%) |
| Pathologic stage |  |  |  |  |  |  |  |
| 2c | 4 (20%) | 3 (20%) | 4 (14.8%) | 6 (30%) | 0.37** | 0.22** | 0.22** |
| 3a | 10 (50%) | 9 (60%) | 15 (55.5%) | 10 (50%) |
| 3b | 6 (30%) | 3 (20%) | 8 (29.6%) | 4 (20%) |
| Positive nodes | 2 (10%) | 2 (10%) | 4 (14.8%) | 3 (15%) | 1.00*** | 1.00*** | 1.00*** |
| Positive margins | 5 (25%) | 5 (25%) | 5 (18.5%) | 8 (40%) | 0.50*** | 0.18*** | 0.18*** |

IHC = immunohistochemistry; mRNA = messenger RNA; PSA = prostate-specific antigen.

* Mann-Whitney.

** χ^2^ test.

***Fisher’s exact test.

† Postprostatectomy Gleason score must be interpreted with caution because prostate cancer architecture and thus Gleason score are affected by hormonal therapy, even if short lived in this study. Mild architectural changes were observed in the post-treatment group. No patients received any hormonal treatment of 5α-reductase inhibitor other than degarelix within 6 mo prior to undergoing radical prostatectomy.

**Supplementary Table 2 – Gene expression levels of significantly upregulated genes in human prostate cancer following degarelix treatment (adjusted *p* value <0.05)**

| Symbol reannotated | Relative expression | Adjusted *p* value |
| --- | --- | --- |
| ANGPTL4 | 2.064383615 | 0.01039987 |
| SLPI | 2.033023076 | 0.003128342 |
| ERP27 | 2.012432172 | 0.000501987 |
| NFKBIZ | 1.909069438 | 0.041148152 |
| RARRES1 | 1.862223943 | 0.043306467 |
| CDH3 | 1.860709557 | 0.000101754 |
| SYTL2 | 1.843680841 | 0.00527852 |
| GOLGA8B | 1.815532567 | 0.01696905 |
| GRP | 1.80925527 | 0.028875768 |
| MFAP4 | 1.792305453 | 0.011900729 |
| PDE5A | 1.785283891 | 0.020737466 |
| DACT2 | 1.782757811 | 0.006197525 |
| GPR116 | 1.75635939 | 0.004215848 |
| ATHL1 | 1.724975619 | 0.00174522 |
| TYMP | 1.677911751 | 0.019652703 |
| TSC22D1 | 1.668446458 | 0.001230571 |
| EOMES | 1.66601051 | 0.00220648 |
| LY96 | 1.662103519 | 0.001615205 |
| GRAMD4 | 1.636066879 | 0.012891544 |
| CDK19 | 1.632833522 | 0.022744939 |
| C2CD4B | 1.625767148 | 0.012112494 |
| GBP2 | 1.620314632 | 0.012374578 |
| MPZL2 | 1.618674284 | 0.001276651 |
| ACSL5 | 1.615969418 | 0.048042948 |
| ARHGEF2 | 1.575826117 | 0.00527852 |
| HAVCR2 | 1.566454071 | 0.003719722 |
| VSTM2L | 1.562419361 | 0.04904607 |
| SLC16A3 | 1.558349387 | 0.021125672 |
| RHPN2 | 1.557900638 | 0.012943345 |
| DKFZp434K1323 | 1.557192244 | 0.040457768 |
| JMJD7-PLA2G4B | 1.552573771 | 0.013007916 |
| REXO2 | 1.551404962 | 0.009349945 |
| IL18BP | 1.550472204 | 0.026562657 |
| FAM46A | 1.546643942 | 0.000801785 |
| ESR1 | 1.533580458 | 0.000216695 |
| IGFBP4 | 1.533018689 | 0.015898287 |
| C7orf58 | 1.531844585 | 0.021276239 |
| BRSK1 | 1.527855636 | 0.048266255 |
| LRP5 | 1.523982234 | 0.000174766 |
| CEBPB | 1.510920678 | 0.006396208 |
| SCN2A | 1.506392932 | 0.020485671 |
| TMC5 | 1.504827513 | 0.031566402 |
| TOX2 | 1.502336168 | 0.015502823 |
| NPTX1 | 1.501635759 | 0.014162332 |
| AIF1 | 1.500090429 | 0.031569273 |
| AR | 1.498121917 | 0.006723596 |
| CEBPD | 1.497310715 | 0.035267423 |
| CDK5RAP3 | 1.496456422 | 0.00133824 |
| MLLT6 | 1.490164902 | 0.000478442 |
| WASH2P | 1.484259785 | 0.028291613 |
| SNORD56 | 1.483232722 | 0.002042936 |
| ABCA1 | 1.480822828 | 0.014162332 |
| KAT2A | 1.479228156 | 0.003658208 |
| MST1 | 1.479163579 | 0.003727373 |
| SLC11A2 | 1.478188284 | 0.01249302 |
| ABCC5 | 1.477806449 | 0.000320196 |
| ANTXR1 | 1.474851775 | 0.024496224 |
| SNCAIP | 1.471105469 | 0.014695514 |
| MID1 | 1.470694929 | 0.000320196 |
| PABPC1L | 1.469244915 | 0.015303581 |
| EHBP1L1 | 1.45692951 | 0.018938432 |
| ITGAX | 1.455828282 | 0.012928507 |
| FGR | 1.453629525 | 0.026214051 |
| ALPL | 1.452727637 | 0.013189186 |
| ARGLU1 | 1.452549509 | 0.013068848 |
| SNRNP70 | 1.450266788 | 0.021461142 |
| MAN2C1 | 1.449682039 | 8.65E-05 |
| PARP3 | 1.444695007 | 0.000237399 |
| PTGES | 1.444626792 | 0.033973641 |
| C19orf66 | 1.443734209 | 0.000785745 |
| DENND4B | 1.441078004 | 0.002386812 |
| LGALS8 | 1.43795957 | 0.002050752 |
| C2CD4B | 1.43603586 | 0.025699964 |
| IL4R | 1.432454142 | 0.024497377 |
| AMT | 1.432197639 | 0.003698779 |
| HDC | 1.430924411 | 0.04131591 |
| NPW | 1.426196936 | 0.000796642 |
| EFNA1 | 1.42618156 | 0.021711735 |
| ZNF266 | 1.422569442 | 0.003994954 |
| RALGDS | 1.420357154 | 0.021295843 |
| N6AMT1 | 1.420286123 | 0.00430092 |
| FBXO32 | 1.420149135 | 0.021295843 |
| OGT | 1.417754942 | 0.010435124 |
| RAB23 | 1.415914855 | 0.02860102 |
| SNORD68 | 1.412911108 | 0.007768646 |
| RBM25 | 1.412712898 | 0.006113972 |
| GRP | 1.411549854 | 0.026836192 |
| SIRPA | 1.410032123 | 0.008748175 |
| PKDCC | 1.409718872 | 0.014819436 |
| MAP3K5 | 1.408783904 | 0.021569211 |
| FLJ35776 | 1.408467016 | 8.66E-05 |
| USF1 | 1.408444337 | 0.006464976 |
| PAM | 1.405802876 | 0.003994954 |
| CXCL16 | 1.405013113 | 0.008819737 |
| RAB31 | 1.402751042 | 0.013948074 |
| SLC1A3 | 1.402609277 | 0.009315835 |
| LEPREL2 | 1.401756309 | 0.004933344 |
| MYOZ3 | 1.399823202 | 0.024686187 |
| PROS1 | 1.399138416 | 0.008555055 |
| LEMD3 | 1.395600725 | 0.026128385 |
| LOC388152 | 1.39465091 | 0.042752202 |
| C6orf192 | 1.392402519 | 0.005047771 |
| NXF1 | 1.390522627 | 0.000320196 |
| DUSP22 | 1.389909265 | 4.90E-05 |
| KRBA1 | 1.389672694 | 0.004114585 |
| SNORD83B | 1.389065132 | 0.013007916 |
| SH3GLB2 | 1.387413353 | 0.00177805 |
| CXXC1 | 1.382584273 | 0.003719722 |
| ABCC5 | 1.381816544 | 0.004879493 |
| PPP2R2A | 1.379835292 | 0.000785745 |
| NTN4 | 1.379479141 | 0.02609049 |
| RAI14 | 1.376980683 | 0.003376668 |
| PCBP2 | 1.376739245 | 0.001513672 |
| VARS2 | 1.375512658 | 0.017124452 |
| SNRNP70 | 1.37368329 | 0.020161293 |
| CSDC2 | 1.372118651 | 0.011360534 |
| THUMPD2 | 1.370286375 | 0.001202142 |
| TMEM173 | 1.368761119 | 0.007873656 |
| CCDC93 | 1.367154252 | 0.000851853 |
| IFITM2 | 1.366751717 | 0.032058269 |
| NPEPL1 | 1.36411416 | 0.002029037 |
| LOC155060 | 1.363477429 | 0.039398483 |
| CENPV | 1.362341541 | 0.002545264 |
| ST5 | 1.362110446 | 0.021420809 |
| IFNGR1 | 1.361512442 | 0.021367831 |
| ZNF263 | 1.36064556 | 3.35E-06 |
| UNQ5808 | 1.35969288 | 0.002949538 |
| SNORD66 | 1.358662255 | 0.005041983 |
| BAZ2B | 1.357721506 | 0.011081309 |
| SNORD95 | 1.35486156 | 0.003698779 |
| RSL24D1 | 1.353548566 | 0.000251821 |
| AKAP8L | 1.353101891 | 0.02453605 |
| TUBB2B | 1.352331721 | 0.014098652 |
| PKD1 | 1.350932442 | 0.009207759 |
| CCDC130 | 1.350781753 | 0.002050752 |
| C5orf30 | 1.346610204 | 0.015286608 |
| HSPA12A | 1.344067105 | 0.015648849 |
| FOXA2 | 1.343936181 | 0.013233379 |
| CEP164 | 1.343642053 | 0.002837138 |
| NXF1 | 1.342364048 | 0.000653982 |
| STAB1 | 1.34233948 | 0.0123632 |
| PRPF3 | 1.341796904 | 0.000411446 |
| BRD9 | 1.341438424 | 0.004879493 |
| DMBT1 | 1.340492706 | 0.011033537 |
| OLR1 | 1.340003065 | 0.032553127 |
| PRR3 | 1.339527157 | 0.000984068 |
| OLFML2B | 1.337985995 | 0.009147212 |
| CNN3 | 1.337364408 | 0.032831721 |
| CPT1B | 1.336627471 | 0.011228688 |
| ATP9A | 1.336291847 | 0.004008349 |
| LTBP3 | 1.336125965 | 0.021286783 |
| SNHG1 | 1.33490019 | 0.019335047 |
| IMPDH2 | 1.33466992 | 0.011006194 |
| CD520639 | 1.333199064 | 0.026288202 |
| RCC1 | 1.333114058 | 0.002050752 |
| STAT2 | 1.332781652 | 0.005391761 |
| HS3ST2 | 1.332558542 | 0.041461599 |
| SNORD46 | 1.332323311 | 0.00354485 |
| BOD1 | 1.332209182 | 1.85E-05 |
| CLK2 | 1.331612979 | 0.000320196 |
| BX104024 | 1.330552324 | 0.000237399 |
| AHI1 | 1.330430274 | 0.033973641 |
| RBM22 | 1.329558258 | 8.34E-06 |
| SCNN1D | 1.328320801 | 0.017409331 |
| VIPR2 | 1.326776817 | 0.015609234 |
| ABTB1 | 1.326448642 | 0.01100682 |
| MSN | 1.326418623 | 0.040215591 |
| TTC39C | 1.325023435 | 0.016664522 |
| TMEM106C | 1.323058694 | 0.014162332 |
| OBFC2A | 1.322650779 | 0.013233379 |
| KIAA0907 | 1.321218276 | 0.014695514 |
| LOC401308 | 1.320519654 | 0.018981174 |
| NCS1 | 1.320272224 | 0.045095974 |
| TBX3 | 1.319969229 | 0.016777678 |
| BHLHE41 | 1.319925593 | 0.026128385 |
| RAB3IL1 | 1.319658634 | 0.037900098 |
| BZRAP1 | 1.318674446 | 0.007148346 |
| CAPZA2 | 1.318033503 | 0.016625329 |
| MVP | 1.317815613 | 0.012638905 |
| ZBTB48 | 1.317540653 | 0.027671594 |
| LAT | 1.31707262 | 0.013542121 |
| THADA | 1.316118301 | 0.008484896 |
| SHB | 1.315925594 | 0.014057792 |
| STEAP3 | 1.315325989 | 0.017657239 |
| STAT3 | 1.315202432 | 0.00745268 |
| SLC4A7 | 1.314905377 | 0.039871981 |
| PLEKHA4 | 1.314858294 | 0.035264543 |
| PER2 | 1.31457071 | 0.023852908 |
| BTAF1 | 1.31369113 | 0.010511154 |
| SGSM1 | 1.312061507 | 0.001449121 |
| ERCC5 | 1.312028165 | 0.027671594 |
| SNORD96A | 1.311525177 | 0.004116571 |
| FES | 1.311431492 | 0.004338334 |
| PTCHD1 | 1.309404624 | 0.036589452 |
| SRSF6 | 1.307328335 | 0.011093013 |
| ST6GALNAC6 | 1.306688804 | 0.033468299 |
| HIST1H2BF | 1.305730093 | 0.016664522 |
| KDM2B | 1.303268493 | 0.023212086 |
| C20orf20 | 1.301406826 | 0.018653661 |
| P2RY11 | 1.300289132 | 0.004925839 |
| RBM5 | 1.299070361 | 0.006136951 |
| WDR74 | 1.298977741 | 0.018156492 |
| TMEM51 | 1.298500197 | 0.033350044 |
| LYSMD4 | 1.29759862 | 0.001609792 |
| CAMLG | 1.297024537 | 0.000251821 |
| PLEKHN1 | 1.296102983 | 0.018927545 |
| RGMA | 1.295308626 | 0.010881747 |
| ZMYND15 | 1.294984149 | 0.011159187 |
| NFX1 | 1.294611487 | 0.018532163 |
| PSMB10 | 1.294398474 | 0.024474495 |
| PCNX | 1.294184482 | 0.007165272 |
| PKDCC | 1.292793969 | 0.016777678 |
| TFAM | 1.291986346 | 0.003031269 |
| CTPS | 1.291934117 | 0.024431293 |
| TRMT61A | 1.291730738 | 0.008931063 |
| PGM2 | 1.291063217 | 0.038863038 |
| TOX2 | 1.290479956 | 0.035124029 |
| SUZ12 | 1.289865148 | 0.011026434 |
| NPTN | 1.288917228 | 0.026291957 |
| TMUB2 | 1.288673137 | 0.028428495 |
| YTHDC1 | 1.288338542 | 0.000785745 |
| HAMP | 1.288132673 | 0.007262539 |
| NUMA1 | 1.286817241 | 0.025372964 |
| RBM14 | 1.286381923 | 0.001384552 |
| RCC1 | 1.285239355 | 0.011757369 |
| SNORD38A | 1.285053422 | 0.002310694 |
| BCL2 | 1.284433112 | 0.033195165 |
| POLRMT | 1.284357737 | 0.00133488 |
| ZNF337 | 1.283921797 | 0.016777678 |
| APBB3 | 1.28365569 | 0.011234264 |
| DMKN | 1.283532658 | 0.004933344 |
| ZSWIM6 | 1.283376969 | 0.027317523 |
| ANTXR1 | 1.282654611 | 0.009164983 |
| RFX2 | 1.282253188 | 0.011081309 |
| DKFZp434K1323 | 1.281683615 | 0.049443971 |
| AP1S2 | 1.281555691 | 0.03576109 |
| ANAPC4 | 1.280998695 | 0.01722523 |
| SNORD89 | 1.280728935 | 0.021276239 |
| SH3BP5L | 1.280653186 | 0.030415832 |
| FLOT1 | 1.280238317 | 0.001271345 |
| ATG16L2 | 1.280229641 | 0.004882569 |
| GDPD3 | 1.280132021 | 0.007258832 |
| CCNL2 | 1.279659708 | 0.010960017 |
| SS18L2 | 1.279380367 | 0.017124452 |
| DNAJA2 | 1.278994629 | 0.002375176 |
| TMEM50B | 1.278448724 | 0.002232401 |
| AKAP13 | 1.278393225 | 0.013948074 |
| LGALS8 | 1.277864914 | 0.019919623 |
| PCM1 | 1.275967415 | 0.019642805 |
| TEF | 1.275780883 | 0.045095974 |
| IL13RA1 | 1.275518316 | 0.038064177 |
| PDGFRL | 1.275257019 | 0.013233379 |
| SYK | 1.275191343 | 0.029712605 |
| KIAA0700 | 1.274643889 | 0.001304032 |
| FAM113B | 1.274472297 | 0.021295843 |
| TTC21A | 1.274086578 | 0.031772402 |
| DDX39B | 1.273880469 | 0.017901484 |
| RPS6KB1 | 1.273351214 | 0.002969485 |
| GATS | 1.273304987 | 0.019573988 |
| CLSTN2 | 1.273161299 | 0.019613616 |
| LYRM1 | 1.273127949 | 0.003332027 |
| UBAP2L | 1.273056191 | 0.00416849 |
| ARHGEF3 | 1.27270498 | 0.040590068 |
| SRRT | 1.272467426 | 0.006340394 |
| OGFR | 1.271726869 | 0.002277593 |
| PARL | 1.271286002 | 0.000236068 |
| SSBP2 | 1.270381343 | 0.018830763 |
| CD86 | 1.269766066 | 0.049657941 |
| THBS3 | 1.269546611 | 0.028517232 |
| ANKRD50 | 1.269508471 | 0.021276101 |
| LRRC49 | 1.266245485 | 0.000653982 |
| SETDB2 | 1.265323276 | 0.034256507 |
| CHD4 | 1.265249971 | 0.008962494 |
| CYP21A1P | 1.264832789 | 0.006750946 |
| SUPT5H | 1.264795044 | 0.005236734 |
| NFKB1 | 1.264611091 | 0.007480933 |
| PLXNA1 | 1.264597655 | 0.022221063 |
| NCOA1 | 1.264433708 | 0.02878188 |
| dJ1198O21.1 | 1.264204796 | 0.017124452 |
| PI4KB | 1.264040747 | 0.016664522 |
| PDE1A | 1.263774392 | 0.045911014 |
| CENPV | 1.263718279 | 0.003031269 |
| AGPAT9 | 1.263145301 | 0.036730252 |
| GABBR1 | 1.263131897 | 0.045911014 |
| ABTB1 | 1.262518394 | 0.001212645 |
| TLE2 | 1.262473318 | 0.022619693 |
| TRMT1 | 1.262141538 | 0.033032324 |
| RPUSD4 | 1.261029787 | 0.00527852 |
| BRD2 | 1.260904595 | 0.00885098 |
| RPF2 | 1.260814021 | 0.048652855 |
| IRF3 | 1.260733747 | 0.016530506 |
| CRY2 | 1.259111162 | 0.004008349 |
| NEURL4 | 1.259091734 | 0.013233379 |
| MIB2 | 1.258571198 | 0.037292531 |
| C17orf45 | 1.258092316 | 0.027156127 |
| BRPF1 | 1.25773347 | 0.005059606 |
| PABPN1 | 1.256718441 | 0.041865579 |
| MRPL2 | 1.256013039 | 0.000984446 |
| CCDC45 | 1.255975416 | 0.043563938 |
| CP110 | 1.255104457 | 0.032831721 |
| PARP3 | 1.254438192 | 0.002141938 |
| PTPRA | 1.252991273 | 0.014057792 |
| GNPDA1 | 1.252512245 | 0.00177805 |
| MATR3 | 1.252358071 | 0.000984446 |
| HSPC072 | 1.25195625 | 0.018127467 |
| LTA4H | 1.251774878 | 0.002207996 |
| SDAD1 | 1.251243806 | 0.00527852 |
| PHF21A | 1.251078559 | 0.022020298 |
| RNF207 | 1.25099741 | 0.011900729 |
| EDC4 | 1.250601187 | 0.016707555 |
| C19orf2 | 1.249896116 | 0.009245119 |
| SNX22 | 1.249724206 | 0.00422611 |
| RNF149 | 1.248527937 | 0.002135465 |
| SNORA57 | 1.248506115 | 0.008875324 |
| CSNK1E | 1.247541534 | 0.004729531 |
| SPG7 | 1.247311725 | 0.046045137 |
| YWHAH | 1.247267427 | 0.047540178 |
| CDKN2AIP | 1.24693012 | 0.011589853 |
| HDAC2 | 1.246854338 | 0.030524447 |
| PLCB2 | 1.246203018 | 0.045626417 |
| BATF3 | 1.245703836 | 0.026138343 |
| PARL | 1.244811058 | 0.001631441 |
| NAT10 | 1.24372983 | 0.000461711 |
| SNRK | 1.243286521 | 0.021461142 |
| LOC147727 | 1.242988268 | 0.000896583 |
| DHX37 | 1.242918092 | 0.035604877 |
| WDR6 | 1.242888012 | 0.012519677 |
| DUOX1 | 1.242857227 | 0.039889877 |
| LHFP | 1.242813881 | 0.023852908 |
| NUDT3 | 1.242600282 | 0.031092138 |
| UPF2 | 1.242464312 | 0.01696905 |
| CIRH1A | 1.24231188 | 0.019450232 |
| MYT1 | 1.241208457 | 0.013981942 |
| MAMDC4 | 1.241002282 | 0.000422127 |
| NCOA3 | 1.240913825 | 0.021007118 |
| RIOK1 | 1.240402217 | 0.001152486 |
| BCLAF1 | 1.240311401 | 0.035692944 |
| CPNE1 | 1.240232376 | 0.001921816 |
| CTSL1 | 1.239760339 | 0.043434647 |
| YY1AP1 | 1.239743249 | 0.000653982 |
| CDK5RAP3 | 1.239625817 | 0.045911014 |
| DUOXA1 | 1.239568252 | 0.008291519 |
| PDK3 | 1.239438514 | 0.046045137 |
| UPF3B | 1.239192697 | 0.008263014 |
| ATIC | 1.239175411 | 0.003544646 |
| RPL21 | 1.239016549 | 6.53E-05 |
| MAP4K5 | 1.238628939 | 0.021571378 |
| CDK9 | 1.238563151 | 0.002050752 |
| SCRN1 | 1.238014374 | 0.038826356 |
| TPD52L2 | 1.237778422 | 0.006483544 |
| SURF6 | 1.237301207 | 0.029233105 |
| ZNF629 | 1.236778146 | 0.008300161 |
| ADAM7 | 1.236261991 | 0.001449121 |
| NOP16 | 1.236198161 | 0.042680472 |
| MED4 | 1.236092168 | 0.037704265 |
| WDR19 | 1.235852626 | 0.008075634 |
| PTAR1 | 1.235237939 | 0.004600019 |
| PARP1 | 1.234871922 | 0.00243341 |
| SEMA4C | 1.234468488 | 0.016032914 |
| RBM10 | 1.234335683 | 0.037481755 |
| ZNF586 | 1.23429069 | 0.001656347 |
| NUCKS1 | 1.234124167 | 0.043991264 |
| CEPT1 | 1.233795686 | 0.011228688 |
| EIF3D | 1.233018424 | 0.008641291 |
| HEMK1 | 1.232729825 | 0.009894545 |
| RING1 | 1.232683225 | 0.009164983 |
| ARHGEF17 | 1.23185727 | 0.026128385 |
| SHMT1 | 1.231482573 | 0.042052657 |
| HEBP1 | 1.231383916 | 0.019608263 |
| WBSCR20C | 1.231369649 | 0.038068292 |
| ODF3B | 1.231366825 | 0.029582284 |
| AK123101 | 1.231277278 | 0.040590068 |
| GOLGA2L1 | 1.230584058 | 0.000259291 |
| CEP290 | 1.230486748 | 0.0123632 |
| PTCH1 | 1.230377359 | 0.012197311 |
| HIBADH | 1.229959496 | 0.020817721 |
| NAP1L1 | 1.229858481 | 0.02453605 |
| NXPH3 | 1.229770098 | 0.039395343 |
| ZMYM1 | 1.229696471 | 0.004376336 |
| SNX6 | 1.229662671 | 0.018149142 |
| WDR33 | 1.229315831 | 0.013791669 |
| SMAD5 | 1.228866287 | 0.037704265 |
| B4GALNT4 | 1.227880083 | 0.013981942 |
| RPS6KA5 | 1.22754866 | 0.034186288 |
| DQ581928 | 1.227344558 | 0.002541127 |
| RPL21 | 1.227295789 | 0.003698779 |
| ADAR | 1.226988476 | 0.00614934 |
| RPL21 | 1.226744943 | 0.000216695 |
| LOC100133161 | 1.226653255 | 0.021461142 |
| TSPAN15 | 1.225733697 | 0.021276239 |
| NCRNA00173 | 1.22569916 | 0.002247005 |
| NCRNA00095 | 1.225653101 | 0.043393904 |
| WDR46 | 1.225343868 | 0.004338334 |
| ATP6V1E1 | 1.225212772 | 0.005526016 |
| WDR27 | 1.22429354 | 0.032924826 |
| DUSP22 | 1.224110569 | 0.002890051 |
| MCM7 | 1.223862754 | 0.021705117 |
| SNORD110 | 1.22377958 | 0.008105328 |
| RPUSD2 | 1.223729913 | 0.00527852 |
| MGC57346 | 1.223490758 | 0.001631441 |
| C7orf58 | 1.223396251 | 0.035708694 |
| ZNF540 | 1.221230798 | 0.020161293 |
| APOC4-APOC2 | 1.221205422 | 0.003544646 |
| LRP5 | 1.220882745 | 0.000251951 |
| HNRNPD | 1.220492648 | 0.002232401 |
| BX647159 | 1.220388882 | 0.042155657 |
| SAAL1 | 1.220361928 | 0.035659367 |
| UBE2G2 | 1.219973967 | 0.008819737 |
| SEMA6C | 1.219973686 | 0.000855013 |
| MLL4 | 1.219356394 | 0.014237366 |
| DIMT1L | 1.218798445 | 0.023852908 |
| ZNF26 | 1.218709148 | 0.004879493 |
| MPP1 | 1.218543962 | 0.009920477 |
| PRKDC | 1.218338878 | 0.002141938 |
| RHOT2 | 1.218193415 | 0.014803304 |
| E2F3 | 1.217639211 | 0.039327317 |
| METAP2 | 1.217566897 | 0.007365119 |
| WDR59 | 1.217352857 | 0.013007916 |
| DKC1 | 1.217330705 | 0.002277593 |
| NIPBL | 1.217258722 | 0.006136951 |
| DDX42 | 1.217058816 | 0.003716622 |
| ANKRD6 | 1.216332837 | 0.014594374 |
| SETD1A | 1.215938563 | 0.009315835 |
| GRAMD2 | 1.215871815 | 0.00401477 |
| PRRC2A | 1.215753865 | 0.021276239 |
| TAZ | 1.215328796 | 0.008748175 |
| MGEA5 | 1.21482492 | 0.02646569 |
| LTV1 | 1.214261316 | 0.034454374 |
| NOL6 | 1.214184898 | 0.032157129 |
| SIRT5 | 1.21416677 | 0.010995831 |
| ZCWPW1 | 1.214065206 | 0.015317002 |
| RFX2 | 1.213483278 | 0.028428495 |
| DHX40 | 1.212631682 | 0.016183639 |
| EEF1B2 | 1.212538262 | 0.014695514 |
| HSD3B7 | 1.211123045 | 0.030704479 |
| NOL8 | 1.210547492 | 0.034174553 |
| RNF31 | 1.210499278 | 0.021450537 |
| SOX21 | 1.210184305 | 0.031106432 |
| FAM184A | 1.210103233 | 0.027184609 |
| LOC91316 | 1.209904735 | 0.004939812 |
| SYTL2 | 1.209873933 | 0.004275787 |
| KLF13 | 1.209691596 | 0.048300568 |
| FAM160A2 | 1.209399618 | 0.017467175 |
| PACRG | 1.209329684 | 0.003719722 |
| C7orf40 | 1.209112771 | 0.022189641 |
| ZNF302 | 1.208751274 | 0.030107918 |
| NUDCD3 | 1.208533819 | 0.025905181 |
| PPM1M | 1.208484925 | 0.047628788 |
| PHF3 | 1.208432032 | 0.008300161 |
| DMKN | 1.208390693 | 0.009656929 |
| C12orf41 | 1.207966831 | 0.019450232 |
| SNORD25 | 1.207885049 | 0.000904601 |
| BANP | 1.207818233 | 0.021007118 |
| MBD6 | 1.207758329 | 0.035659367 |
| PRKAA1 | 1.207499916 | 0.012877473 |
| POLRMT | 1.206930054 | 0.021276239 |
| FLJ45244 | 1.206830029 | 0.014162332 |
| LOC286367 | 1.206816007 | 0.03796284 |
| LOC285300 | 1.206155335 | 0.018750811 |
| C7orf58 | 1.206154454 | 0.002247005 |
| UBE3A | 1.205969021 | 0.028005668 |
| PQLC1 | 1.205385086 | 0.025488415 |
| RSBN1 | 1.20524515 | 0.017854147 |
| LRRC37B | 1.205118493 | 0.036359887 |
| OBSCN | 1.204119859 | 0.022068522 |
| DMAP1 | 1.203728339 | 0.043933334 |
| METTL1 | 1.203362492 | 0.041327558 |
| WDR75 | 1.203298274 | 0.027483899 |
| FAM164C | 1.203263733 | 0.027038219 |
| PKD1 | 1.203140802 | 0.021295843 |
| ZNF395 | 1.202286196 | 0.041461599 |
| LOC80154 | 1.202244614 | 0.049497927 |
| MYST1 | 1.201600745 | 0.021219341 |
| SRSF7 | 1.201443828 | 0.037840539 |
| EPOR | 1.201239816 | 0.00527852 |
| FBRSL1 | 1.199917841 | 0.00812633 |
| SMEK1 | 1.199765894 | 0.036572889 |
| CCS | 1.199671614 | 0.011081309 |
| PAN2 | 1.199223671 | 0.035286369 |
| HDAC7 | 1.199212181 | 0.04131591 |
| EEF1D | 1.198734644 | 0.017669904 |
| C2orf42 | 1.19856195 | 0.011410116 |
| C1orf228 | 1.198028734 | 0.014162332 |
| TAF15 | 1.197282743 | 0.015714425 |
| TOMM7 | 1.197248007 | 0.000216695 |
| MXRA8 | 1.196869134 | 0.045626417 |
| ARHGAP17 | 1.196856789 | 0.00484359 |
| PHF1 | 1.196679063 | 0.00177805 |
| UCKL1 | 1.196416679 | 0.04346981 |
| GTF2E2 | 1.196165705 | 0.014819436 |
| RPL29 | 1.196034126 | 0.041148152 |
| DIS3L | 1.194998794 | 0.045595613 |
| ETF1 | 1.193984805 | 0.022670328 |
| SLC20A2 | 1.193676844 | 0.02895037 |
| ATG14 | 1.193570765 | 0.022068522 |
| TMEM69 | 1.192702137 | 0.036506564 |
| ATF6B | 1.192535869 | 0.005008459 |
| PRR14 | 1.192522353 | 0.048300568 |
| CD300LF | 1.19190679 | 0.037307466 |
| DTX3 | 1.191612269 | 0.019725475 |
| POLR1D | 1.191266446 | 0.044881724 |
| MED12 | 1.191194248 | 0.008819737 |
| PPP2CA | 1.191176559 | 0.033746367 |
| CRBN | 1.190712441 | 0.02417902 |
| METTL17 | 1.190692686 | 0.031101774 |
| RPL17 | 1.190675481 | 0.021571378 |
| USP46 | 1.190139365 | 0.00368918 |
| RFX7 | 1.19004696 | 0.026545484 |
| HEATR6 | 1.189813773 | 0.019642805 |
| STOML2 | 1.18967667 | 0.000173222 |
| SNHG10 | 1.189615414 | 0.000251821 |
| CD300A | 1.189526906 | 0.01696905 |
| GRB7 | 1.189324688 | 0.022235123 |
| ILKAP | 1.189108321 | 0.032568602 |
| LOC284023 | 1.189087253 | 0.019859843 |
| CCDC59 | 1.188919929 | 0.025905181 |
| TRIM39 | 1.188661003 | 0.01022877 |
| PRPF4 | 1.188290898 | 0.007365119 |
| RPL21 | 1.188243625 | 2.20E-05 |
| FBL | 1.188153984 | 0.03647484 |
| EMD | 1.188081981 | 0.003842245 |
| CHTOP | 1.187949912 | 0.007212646 |
| CEPT1 | 1.187792743 | 0.044672331 |
| NCRNA00092 | 1.187450708 | 0.004435265 |
| RIN3 | 1.187417135 | 0.011081309 |
| ZC3HC1 | 1.18740004 | 0.000820377 |
| ZNF529 | 1.187342773 | 0.019652703 |
| RBCK1 | 1.186988759 | 0.036496071 |
| C4orf11 | 1.186809695 | 0.008509185 |
| AVP | 1.186574188 | 0.018719986 |
| IPO11 | 1.186469809 | 0.009920477 |
| SQSTM1 | 1.186296276 | 0.029448121 |
| SRPK3 | 1.185962129 | 0.045911014 |
| CORO7 | 1.185662965 | 0.047628788 |
| RPL21 | 1.18552172 | 8.66E-05 |
| STX1A | 1.185488505 | 1.73E-05 |
| VASH1 | 1.18461465 | 0.025232597 |
| KIAA1967 | 1.184437045 | 0.025228697 |
| LOC349114 | 1.184300757 | 0.028288609 |
| SMARCD1 | 1.184230259 | 0.009094054 |
| HIST2H2BF | 1.184223789 | 0.04131591 |
| LAT | 1.183858537 | 0.02269419 |
| RASA1 | 1.183820772 | 0.045297479 |
| SNORD31 | 1.183734876 | 0.005790025 |
| ORAOV1 | 1.183577993 | 0.047004152 |
| TMEM194A | 1.183483147 | 0.036921826 |
| RIN1 | 1.183209481 | 0.042384422 |
| TTLL5 | 1.183078337 | 0.006136951 |
| ARAP1 | 1.182993233 | 0.033085998 |
| AKAP13 | 1.182854064 | 0.006233348 |
| SNORA10 | 1.182587934 | 0.022949543 |
| ARHGEF12 | 1.18232732 | 0.014694147 |
| PHC1 | 1.18197301 | 0.001108986 |
| MSH5 | 1.181803379 | 0.004714015 |
| FAM193B | 1.181099963 | 0.032168809 |
| NOXA1 | 1.180821699 | 0.039395343 |
| ZNF317 | 1.180488194 | 0.011488909 |
| SLCO1B3 | 1.180246438 | 0.000505728 |
| BUB3 | 1.179947212 | 0.022020298 |
| ASPHD1 | 1.179707341 | 0.027483899 |
| SF3A3 | 1.179419118 | 0.038467656 |
| ZNF788 | 1.179219689 | 0.011228688 |
| 3-11 | 1.178845453 | 0.002078917 |
| NBEAL2 | 1.178807444 | 0.043788001 |
| TRAF2 | 1.178565876 | 0.033793533 |
| ZDHHC11 | 1.178221488 | 0.001386354 |
| C1orf203 | 1.177880242 | 0.03626086 |
| DHX30 | 1.177578065 | 0.010511862 |
| C12orf47 | 1.177268476 | 0.002242081 |
| ZNF133 | 1.176375089 | 0.042873015 |
| METTL17 | 1.176302244 | 0.017653495 |
| SFSWAP | 1.17605267 | 0.027755545 |
| CPLX1 | 1.175950149 | 0.015502823 |
| PPP2R5A | 1.175853411 | 0.045959677 |
| PTK2B | 1.175827425 | 0.035562519 |
| LOC729234 | 1.175441565 | 0.006544105 |
| SLC25A14 | 1.175399903 | 0.003698779 |
| C19orf43 | 1.175175327 | 0.008741365 |
| ART3 | 1.175020473 | 0.037992591 |
| LOC390940 | 1.174578635 | 0.017824952 |
| LOC349114 | 1.174125164 | 0.009147212 |
| DIABLO | 1.174005354 | 0.0305564 |
| FREM1 | 1.17368839 | 0.00177805 |
| HUAT | 1.173460522 | 0.033792853 |
| SNORD12 | 1.172793707 | 0.006197525 |
| ZCCHC7 | 1.172348056 | 0.021705117 |
| BLMH | 1.172111553 | 0.004879493 |
| CRIPT | 1.171833502 | 0.029192476 |
| TFIP11 | 1.171335451 | 0.004437833 |
| PRR3 | 1.171309183 | 0.00527852 |
| RPL21 | 1.171179767 | 0.000188542 |
| TTC27 | 1.170046028 | 0.036319856 |
| TTC39C | 1.169385195 | 0.043434647 |
| WDR5 | 1.169098079 | 0.044636443 |
| CROCC | 1.168867114 | 0.027648018 |
| ZNF322 | 1.168786351 | 0.006197525 |
| UNQ2487 | 1.168280065 | 0.033350044 |
| SUPT16H | 1.168110327 | 0.013542121 |
| ARMCX5 | 1.168037041 | 0.037292531 |
| GON4L | 1.167634777 | 0.043760398 |
| GLTSCR2 | 1.167626911 | 0.03903592 |
| GAS2 | 1.167448716 | 0.00202832 |
| XPC | 1.167443118 | 0.038205653 |
| BU659183 | 1.167251159 | 0.009717463 |
| HEATR8-TTC4 | 1.166936148 | 0.010120871 |
| MED28 | 1.166826607 | 0.039420972 |
| GMEB1 | 1.166621603 | 0.022262207 |
| DHX8 | 1.166487002 | 0.002232643 |
| AK126437 | 1.16634287 | 0.00614934 |
| SLMO1 | 1.166247514 | 0.008748175 |
| INPP5B | 1.16620246 | 0.014229326 |
| LOC349114 | 1.166005135 | 0.038264431 |
| OSCAR | 1.165983053 | 0.046748297 |
| GNL1 | 1.16580701 | 0.033667816 |
| HIST1H4E | 1.165490146 | 0.044930118 |
| DHX16 | 1.165300745 | 0.00123277 |
| RALA | 1.165157126 | 0.008291519 |
| KCTD15 | 1.164849009 | 0.02623918 |
| PHF1 | 1.164511066 | 0.019919623 |
| INTU | 1.164261734 | 0.044562438 |
| KIAA0922 | 1.164208366 | 0.035591913 |
| CLASRP | 1.163579808 | 0.036035288 |
| CD1C | 1.163473112 | 0.037641932 |
| GAD1 | 1.162777295 | 0.023852908 |
| LOC100506810 | 1.162500123 | 0.004946737 |
| CHTF18 | 1.162356444 | 0.033973641 |
| SNORD36C | 1.161882715 | 0.015771872 |
| TOMM34 | 1.161819697 | 0.041148152 |
| NDST2 | 1.161579725 | 0.025699964 |
| ACVR2B | 1.161280802 | 0.013233379 |
| HARS2 | 1.161256983 | 0.033897716 |
| RAE1 | 1.160632783 | 0.006146462 |
| CNOT1 | 1.160034908 | 0.024796595 |
| NAP1L4 | 1.160018422 | 0.022240666 |
| DCUN1D4 | 1.159963112 | 0.048042948 |
| ZNF280D | 1.159800169 | 0.045959677 |
| C3orf33 | 1.159686813 | 0.008391043 |
| PCDH19 | 1.159266347 | 0.017467175 |
| ARHGEF1 | 1.159143956 | 0.01022877 |
| AK021761 | 1.158952158 | 0.040590068 |
| SNORA72 | 1.158844303 | 0.04462377 |
| KDM1A | 1.158581173 | 0.016681399 |
| PPOX | 1.158327178 | 0.008248542 |
| LYZ | 1.158294636 | 0.030704479 |
| HIST1H2BM | 1.158267147 | 0.015361584 |
| ZNF212 | 1.158047357 | 0.040215591 |
| BC017676 | 1.158013956 | 0.002541127 |
| SNORD21 | 1.157273022 | 0.005047771 |
| GAR1 | 1.157137351 | 0.021295843 |
| NCOA5 | 1.157018581 | 0.013190584 |
| COL2A1 | 1.156954336 | 0.029098056 |
| RRP8 | 1.156711248 | 0.00614934 |
| ZNF334 | 1.156653211 | 0.045755245 |
| SNORD59B | 1.156330861 | 0.018373974 |
| CDC26 | 1.154945833 | 0.040066619 |
| NOD1 | 1.154833776 | 0.028021036 |
| AP4B1 | 1.154282291 | 0.040590068 |
| PCDHGC3 | 1.154236409 | 0.00363748 |
| RASA4 | 1.15415215 | 0.032568602 |
| RNPS1 | 1.153491252 | 0.011081309 |
| IFT172 | 1.152975326 | 0.010611649 |
| FTSJD2 | 1.152871141 | 0.0305564 |
| LOC346887 | 1.152153865 | 0.018313669 |
| ESPN | 1.15192194 | 0.011081309 |
| TFIP11 | 1.151770453 | 0.014057792 |
| C22orf46 | 1.15157833 | 0.02304065 |
| TOMM7 | 1.151369866 | 0.011104488 |
| TTN | 1.151132532 | 0.000805099 |
| PCSK4 | 1.150938717 | 0.004241216 |
| ABCF1 | 1.150819945 | 0.033116935 |
| SH3KBP1 | 1.150525947 | 0.036730252 |
| DQ581928 | 1.150176999 | 0.016416875 |
| JOSD1 | 1.149806183 | 0.031271057 |
| POLE3 | 1.149764167 | 0.016664522 |
| RPL24 | 1.149508751 | 0.023479138 |
| EPB41L2 | 1.148906701 | 0.048223369 |
| RPL21 | 1.148725361 | 0.000491318 |
| CPZ | 1.148682843 | 0.021383938 |
| MSH5 | 1.148417439 | 0.006082946 |
| RP9 | 1.147906394 | 0.03193541 |
| GRIK1 | 1.147836894 | 0.04723035 |
| CAD | 1.147616923 | 0.002247005 |
| VPS26A | 1.147217862 | 0.044930118 |
| SUPT6H | 1.147051076 | 0.014057792 |
| RGMB | 1.146635309 | 0.02760136 |
| ABCE1 | 1.146577073 | 0.014736696 |
| APTX | 1.146555521 | 0.039015782 |
| SNORD88C | 1.146519225 | 0.027483899 |
| CLK3 | 1.146202414 | 0.021705117 |
| FLJ41484 | 1.14590129 | 0.03881077 |
| LOC100288974 | 1.145848891 | 0.035470214 |
| FLJ45340 | 1.145639884 | 0.017653495 |
| C11orf35 | 1.145554159 | 0.033085998 |
| AKAP8 | 1.145337224 | 0.004618975 |
| L3MBTL2 | 1.145110084 | 0.004933344 |
| FERMT3 | 1.144921532 | 0.021488151 |
| ZNF343 | 1.144641356 | 0.033150145 |
| SLCO3A1 | 1.144546087 | 0.036610639 |
| SSBP4 | 1.144368877 | 0.03796284 |
| LOC349114 | 1.14389185 | 0.018647507 |
| ATXN7L2 | 1.143502921 | 0.045629272 |
| ZNF594 | 1.142937917 | 0.037858241 |
| LOC146481 | 1.142640155 | 0.016245347 |
| ZNF276 | 1.142515048 | 0.007700834 |
| GPN2 | 1.142319514 | 0.032739624 |
| PRDXDD1P | 1.141588618 | 0.009315835 |
| STK11IP | 1.141378431 | 0.029181768 |
| SF3B2 | 1.141224904 | 0.012579098 |
| ANO9 | 1.140823582 | 0.048138853 |
| IL34 | 1.140583203 | 0.00736477 |
| SCN9A | 1.140069644 | 0.008173207 |
| CYB5RL | 1.140046202 | 0.013948074 |
| ACBD4 | 1.139423619 | 0.007657688 |
| E2F6 | 1.13931373 | 0.042384422 |
| RPL35 | 1.138807319 | 0.045095974 |
| SNRP70 | 1.138582483 | 0.02610388 |
| DHX30 | 1.13829828 | 0.015706391 |
| GLMN | 1.138292965 | 0.034116349 |
| NUP188 | 1.137726068 | 0.033192679 |
| PSME1 | 1.137589207 | 0.00174522 |
| ZNF629 | 1.137317474 | 0.022757757 |
| FLJ39639 | 1.137047049 | 0.005350387 |
| CAPN12 | 1.135940031 | 0.03557153 |
| PANK2 | 1.135459081 | 0.02269419 |
| POLR3G | 1.134721617 | 0.01594042 |
| UNC13D | 1.134585468 | 0.020211658 |
| NONO | 1.134337248 | 0.04595859 |
| ARHGEF1 | 1.133834438 | 0.016628989 |
| WDR85 | 1.133371822 | 0.003332027 |
| CALCOCO1 | 1.132721218 | 0.040215591 |
| DDX31 | 1.1322019 | 0.044533539 |
| ZNF419 | 1.131466227 | 0.048619712 |
| NEIL1 | 1.131064552 | 0.01157044 |
| BRD8 | 1.130313778 | 0.024730081 |
| UBE2I | 1.13013715 | 0.032801321 |
| TRIM52 | 1.129539088 | 0.040923335 |
| SNORD85 | 1.128375175 | 0.010511862 |
| DUSP15 | 1.128018396 | 0.02396778 |
| BCMO1 | 1.127506538 | 0.044881724 |
| EFTUD2 | 1.127490857 | 0.049657941 |
| DNAJC8 | 1.126503942 | 0.03161553 |
| SEC31B | 1.126006865 | 0.049195342 |
| WDR70 | 1.125559991 | 0.015342751 |
| HMG20A | 1.124640972 | 0.010093816 |
| MORN1 | 1.124142623 | 0.011360534 |
| IFFO1 | 1.123755488 | 0.020192452 |
| DEPDC5 | 1.122995395 | 0.005215863 |
| POLM | 1.122944411 | 0.047786079 |
| ERI1 | 1.122800027 | 0.02944531 |
| B3GNT8 | 1.122331504 | 0.021313207 |
| MEP1A | 1.122287833 | 0.00046444 |
| SNRPD2 | 1.121492995 | 0.032568602 |
| ZNF335 | 1.120394609 | 0.001147102 |
| CNTROB | 1.120268425 | 0.009717463 |
| RBM39 | 1.120108964 | 0.015373181 |
| BX105743 | 1.120069017 | 0.022371068 |
| GTF3C2 | 1.119914916 | 0.014484709 |
| NCRNA00173 | 1.119163773 | 0.003698779 |
| AL512698 | 1.119094599 | 0.013034466 |
| CDH4 | 1.118468078 | 0.045666334 |
| SYTL3 | 1.118162207 | 0.027671594 |
| VMA21 | 1.11760649 | 0.045629272 |
| LOC349114 | 1.117491876 | 0.015720233 |
| DFNB31 | 1.117395302 | 0.021286783 |
| HPS5 | 1.116744227 | 0.016625329 |
| TAZ | 1.115781793 | 0.029616309 |
| LOC155060 | 1.115444578 | 0.003958205 |
| ZNF707 | 1.115355551 | 0.028921216 |
| AK2 | 1.115109496 | 0.045626417 |
| TET1 | 1.115029392 | 0.041148152 |
| CCL17 | 1.114008413 | 0.004925839 |
| CDK20 | 1.113518634 | 0.037704265 |
| RBMX2 | 1.11309242 | 0.021705117 |
| MRPS10 | 1.112980138 | 0.045875218 |
| L3MBTL1 | 1.112573816 | 0.021276239 |
| ASXL1 | 1.112222425 | 0.02580003 |
| SNORD109B | 1.11207331 | 0.015532586 |
| NKRF | 1.111364097 | 0.011517401 |
| HIST1H2AK | 1.111203979 | 0.001276651 |
| C1orf151 | 1.111190915 | 0.027046154 |
| NFKBIL1 | 1.109456777 | 0.022257603 |
| SNORD62A | 1.109031347 | 0.037481755 |
| RNASEH2C | 1.1088766 | 0.044533539 |
| DAB2IP | 1.108259489 | 0.025905181 |
| MAZ | 1.107974519 | 0.025648037 |
| CD200R1 | 1.107536197 | 0.037270458 |
| PINX1 | 1.107032469 | 0.024952533 |
| C12orf68 | 1.106815017 | 0.017409331 |
| WIZ | 1.106136594 | 0.031335102 |
| SLX1B-SULT1A4 | 1.105445773 | 0.022068522 |
| ZNF512B | 1.104494322 | 0.027671594 |
| SNORD105 | 1.104481218 | 0.003395155 |
| BX104024 | 1.103647643 | 0.003662148 |
| OR51A4 | 1.102865155 | 0.001276651 |
| HIST1H2BD | 1.101953487 | 0.007718107 |
| HTRA4 | 1.100996714 | 0.042218244 |
| LOC645676 | 1.10070017 | 0.026998123 |
| TSNAXIP1 | 1.100609004 | 0.017124452 |
| AK095928 | 1.100199418 | 0.005038445 |
| URB2 | 1.099899791 | 0.038264431 |
| RPL21 | 1.097750723 | 0.003186355 |
| BC094703 | 1.0976062 | 0.022221063 |
| LPPR4 | 1.096907282 | 0.027174737 |
| BX102609 | 1.095368377 | 0.03173912 |
| ANKRD24 | 1.095328149 | 0.048300568 |
| KPNA5 | 1.09419412 | 0.036141617 |
| LOC100133161 | 1.094026421 | 0.018312479 |
| RNF219 | 1.093299959 | 0.040422562 |
| ATP8A2 | 1.093139542 | 0.027602091 |
| VTRNA2-1 | 1.092735276 | 0.044652928 |
| C11orf57 | 1.091865711 | 0.030880673 |
| SHC4 | 1.091424129 | 0.022115728 |
| NTN5 | 1.091385077 | 0.046830081 |
| DKFZp686K1684 | 1.091346703 | 0.038320188 |
| IGFN1 | 1.090614268 | 0.009656929 |
| C4orf46 | 1.090607126 | 0.014057792 |
| WWC2 | 1.090219899 | 0.039395343 |
| SNORD65 | 1.090196738 | 0.007753463 |
| SNORD91A | 1.089944664 | 0.004879493 |
| NOVA1 | 1.089270904 | 0.031772402 |
| BUD13 | 1.088605513 | 0.022068522 |
| SIGLEC16 | 1.088206837 | 0.045297479 |
| ZNF300 | 1.088188499 | 0.035374062 |
| LOC100287428 | 1.088173912 | 0.028205169 |
| SNORD74 | 1.0881608 | 0.042867004 |
| SNORA34 | 1.086783807 | 0.021295843 |
| WDR91 | 1.086767369 | 0.014819436 |
| BQ027703 | 1.086603469 | 0.04178199 |
| MUC3A | 1.086527386 | 0.011517401 |
| SCN3A | 1.086054039 | 0.013233379 |
| FLT3 | 1.084905971 | 0.047977232 |
| LCN1 | 1.084443529 | 0.03193541 |
| PCSK6 | 1.084224897 | 0.033973641 |
| FAM156B | 1.084139569 | 0.02453605 |
| TMPRSS3 | 1.084109676 | 0.010995831 |
| ZNF571 | 1.083812763 | 0.018653661 |
| OR56A4 | 1.083565146 | 0.002706304 |
| LOC348840 | 1.083534312 | 0.031133927 |
| C12orf68 | 1.082955546 | 0.020557243 |
| C21orf130 | 1.082635819 | 0.049958337 |
| OOEP | 1.081185702 | 0.030828237 |
| SFRP5 | 1.080610819 | 0.012506307 |
| FAM156A | 1.080058601 | 0.009845468 |
| SNHG12 | 1.079538705 | 0.026545484 |
| LOC401431 | 1.0793743 | 0.039707474 |
| ZDHHC11B | 1.079214812 | 0.011380628 |
| MNX1 | 1.079147683 | 0.036722173 |
| C11orf45 | 1.079042751 | 0.041148152 |
| ANKRD13B | 1.07854749 | 0.04178199 |
| FRMPD2 | 1.078514761 | 0.006197525 |
| CNPY1 | 1.078240282 | 0.02304065 |
| C1orf204 | 1.077971924 | 0.038669601 |
| SIGLEC8 | 1.077351186 | 0.029311479 |
| ANKRD55 | 1.077069623 | 0.027174737 |
| ZSCAN29 | 1.077040205 | 0.022619693 |
| IQCB1 | 1.076283627 | 0.005790025 |
| TMCC2 | 1.07588862 | 0.003128342 |
| AA906426 | 1.075465103 | 0.033973641 |
| AK096576 | 1.075248608 | 0.041461599 |
| OR2A42 | 1.075043894 | 0.008853034 |
| MYH7B | 1.074770823 | 0.035509026 |
| FAM164C | 1.074747918 | 0.045911014 |
| PGLYRP2 | 1.074505798 | 0.027544421 |
| PP1164 | 1.074354463 | 0.032652643 |
| C9orf43 | 1.074276425 | 0.049793941 |
| UBE2I | 1.073699987 | 0.024686187 |
| GRXCR2 | 1.073689344 | 0.035375086 |
| DEFB106B | 1.073257037 | 0.032128271 |
| AMPD3 | 1.072903678 | 0.025692726 |
| BX093041 | 1.072248243 | 0.048223369 |
| ZNF883 | 1.071704954 | 0.04615048 |
| CDAN1 | 1.071596873 | 0.014326927 |
| PSG2 | 1.068867437 | 0.044930118 |
| GALNT10 | 1.068439045 | 0.038998946 |
| DKFZp434B061 | 1.068190553 | 0.013082257 |
| LHFPL3 | 1.068142729 | 0.021295843 |
| TMCO6 | 1.067893728 | 0.027038219 |
| CHRM4 | 1.067584437 | 0.040590068 |
| CECR4 | 1.066394149 | 0.034262218 |
| PNLIPRP1 | 1.06588767 | 0.032652643 |
| CRYGN | 1.065295066 | 0.034499794 |
| DEPDC1B | 1.064263 | 0.036035288 |
| PDGFRB | 1.064097156 | 0.02453605 |
| GAFA3 | 1.063716675 | 0.043250696 |
| COPS2 | 1.063670953 | 0.016664522 |
| C3orf27 | 1.063298833 | 0.046398454 |
| PCSK2 | 1.063250919 | 0.045424831 |
| PSG8 | 1.063148673 | 0.027483899 |
| HIPK3 | 1.063143355 | 0.041148152 |
| C1orf173 | 1.063066302 | 0.036858095 |
| AW592585 | 1.062973479 | 0.041327558 |
| TDGF1 | 1.06296907 | 0.022068522 |
| NOM1 | 1.062651262 | 0.033158809 |
| LOC100131174 | 1.061451189 | 0.042873015 |
| BTF3L1 | 1.061392299 | 0.04138573 |
| POM121C | 1.061360843 | 0.023852908 |
| ZNF41 | 1.061061606 | 0.044366234 |
| DA830074 | 1.061000075 | 0.042752202 |
| MYO3B | 1.060164875 | 0.033667816 |
| LOC399940 | 1.059963804 | 0.042235076 |
| SNORA71D | 1.059439703 | 0.028428495 |
| ZNF708 | 1.059078874 | 0.046980713 |
| RMI1 | 1.058597546 | 0.041327558 |
| SGSM1 | 1.058060938 | 0.039855836 |
| RUFY1 | 1.057860859 | 0.047690253 |
| MASP1 | 1.057563301 | 0.025534431 |
| MMP27 | 1.057294411 | 0.041327558 |
| PCDP1 | 1.056300862 | 0.043847205 |

**Supplementary Table 3 – Gene expression levels of significantly downregulated genes in human prostate cancer following degarelix treatment**

| Symbol reannotated | Relative expression | Adjusted *p* value |
| --- | --- | --- |
| TRH | 0.94585453 | 0.031271057 |
| LOC100132339 | 0.943919593 | 0.049076327 |
| FLJ41327 | 0.943848745 | 0.045297479 |
| PML | 0.943565591 | 0.031772402 |
| FLJ13224 | 0.940656486 | 0.01880443 |
| EIF3C | 0.940222712 | 0.048042948 |
| LOC554223 | 0.939227492 | 0.049976388 |
| PCDHA@ | 0.938486426 | 0.03193541 |
| SPAG11A | 0.937929315 | 0.018981174 |
| LRRC37A4 | 0.937705378 | 0.02210254 |
| ACTR3B | 0.937008846 | 0.047012683 |
| OR9G1 | 0.936156839 | 0.017001773 |
| MYEF2 | 0.935943717 | 0.039420972 |
| PIGP | 0.933739468 | 0.018938432 |
| GMPPB | 0.933482282 | 0.026214051 |
| KIAA1549 | 0.931402795 | 0.043434647 |
| DGKB | 0.931051334 | 0.021705117 |
| GNG10 | 0.929996637 | 0.03881077 |
| METTL11B | 0.928090953 | 0.047977232 |
| KLHL14 | 0.927481712 | 0.048463728 |
| LOC144817 | 0.927037366 | 0.021172122 |
| CELA3A | 0.925168905 | 0.016036656 |
| BU960446 | 0.925137166 | 0.043768272 |
| VWC2 | 0.924830616 | 0.012519677 |
| AK123745 | 0.92480164 | 0.010435124 |
| TMEM150C | 0.923901959 | 0.031029216 |
| BQ006527 | 0.923248926 | 0.034269328 |
| MLLT3 | 0.922895792 | 0.038011485 |
| C18orf16 | 0.921622063 | 0.008819737 |
| AW451125 | 0.919849165 | 0.012516033 |
| PRR13 | 0.919341524 | 0.019923446 |
| STK16 | 0.919096935 | 0.046912127 |
| PRR5 | 0.917425675 | 0.030828237 |
| DGKD | 0.914973664 | 0.02865571 |
| CCT6P3 | 0.914707409 | 0.039395343 |
| LYRM7 | 0.913715554 | 0.043434647 |
| PIGO | 0.91343161 | 0.039420972 |
| SYVN1 | 0.912209437 | 0.042134389 |
| FMN1 | 0.911980048 | 0.000455213 |
| C3orf62 | 0.911541236 | 0.035361426 |
| 01/03/2005 | 0.910031539 | 0.02623918 |
| LOC100129697 | 0.909071681 | 0.03732443 |
| DTYMK | 0.908366571 | 0.042752202 |
| NET1 | 0.907878623 | 0.049076327 |
| C21orf37 | 0.907855037 | 0.018981174 |
| PNPT1 | 0.907342734 | 0.038068292 |
| PCOTH | 0.906624431 | 0.022189641 |
| ZBTB8OS | 0.903277264 | 0.049497411 |
| PTGES3 | 0.903243004 | 0.019642805 |
| AK126511 | 0.902831699 | 0.042463694 |
| GLB1L3 | 0.902821204 | 0.009915181 |
| NIN | 0.902370006 | 0.015898287 |
| KRT8 | 0.901330015 | 0.006146462 |
| C17orf101 | 0.897557363 | 0.049272729 |
| GPR176 | 0.896190041 | 0.005298813 |
| DIP2A | 0.89510856 | 0.035692944 |
| RFXANK | 0.894168155 | 0.044186321 |
| MAP3K2 | 0.893318812 | 0.011510023 |
| TSEN34 | 0.892799496 | 0.013948074 |
| PTPLAD1 | 0.892290759 | 0.026128385 |
| GALR2 | 0.8921681 | 0.003188352 |
| LLGL2 | 0.890443829 | 0.014610185 |
| LENG9 | 0.889748872 | 0.018373974 |
| GPHN | 0.889269617 | 0.048038477 |
| MIER2 | 0.888394641 | 0.044714906 |
| ARID2 | 0.888061286 | 0.027977619 |
| PPIB | 0.88785227 | 0.009147212 |
| BCL2L12 | 0.887279338 | 0.045399939 |
| TRIM3 | 0.887203735 | 0.04492901 |
| BC022382 | 0.88542707 | 0.000573408 |
| MTFP1 | 0.885370001 | 0.024315409 |
| FLJ46111 | 0.882782747 | 0.013776051 |
| PSME3 | 0.882499898 | 0.035375086 |
| CAPN7 | 0.882443018 | 0.011093013 |
| PSEN1 | 0.881656044 | 0.010679251 |
| DNAJB12 | 0.881118606 | 0.017731638 |
| BOLA2B | 0.880622992 | 0.025372964 |
| TSSC1 | 0.879972504 | 0.045647521 |
| STIM2 | 0.87975852 | 0.021383938 |
| GALNS | 0.879640419 | 0.022262207 |
| SRSF10 | 0.879440295 | 0.018332688 |
| LOC643837 | 0.879316712 | 0.024497377 |
| CDCA3 | 0.87849537 | 0.008819737 |
| ADSS | 0.878493737 | 0.033973641 |
| SRPK2 | 0.877909772 | 0.00614934 |
| SENP5 | 0.877850821 | 0.007630105 |
| CLCC1 | 0.877140264 | 0.04478761 |
| KIF20A | 0.875642727 | 0.047416177 |
| EXT2 | 0.875632602 | 0.021717222 |
| AK092218 | 0.875357518 | 0.006396208 |
| WDR26 | 0.875094114 | 0.038246547 |
| ICMT | 0.875074084 | 0.048734522 |
| INO80B-WBP1 | 0.874680022 | 0.038980887 |
| C10orf118 | 0.874433039 | 0.038504789 |
| ZDHHC5 | 0.873582777 | 0.033973641 |
| GPR89B | 0.873532355 | 0.034116349 |
| TMEM17 | 0.87347986 | 0.006483544 |
| GALK2 | 0.873450109 | 0.026214051 |
| TMEM111 | 0.872560715 | 0.038840033 |
| LAMTOR2 | 0.871809717 | 0.032568602 |
| CDT1 | 0.871473801 | 0.033892809 |
| NOMO3 | 0.870416058 | 0.021219341 |
| PVRL2 | 0.870160354 | 0.017963659 |
| ATRN | 0.86957846 | 0.043306467 |
| TM2D1 | 0.869379703 | 0.040590068 |
| ACPL2 | 0.86912552 | 0.030880673 |
| ZBTB8OS | 0.86908172 | 0.024578786 |
| ZNF589 | 0.868502785 | 0.009451092 |
| AF251188 | 0.867411578 | 0.031805711 |
| PSENEN | 0.867356765 | 0.027060048 |
| LMAN2 | 0.866995408 | 0.022913428 |
| BG120325 | 0.866952747 | 0.02318843 |
| PPP2CB | 0.866905897 | 0.009164983 |
| SSNA1 | 0.866750291 | 0.009315835 |
| LYPD6 | 0.866428234 | 0.037481755 |
| ATG4A | 0.866408543 | 0.037388629 |
| LRCH3 | 0.866388064 | 0.029192476 |
| SLC39A3 | 0.865801881 | 0.014819436 |
| BCL2L1 | 0.865518091 | 0.003183961 |
| UNC119 | 0.865064171 | 0.031193596 |
| EXT2 | 0.864913151 | 0.03263729 |
| ADAT1 | 0.864376529 | 0.048266255 |
| KRT18 | 0.864353481 | 0.014609159 |
| RTN2 | 0.864175588 | 0.049586425 |
| PABPC3 | 0.864070818 | 0.033824475 |
| CLSTN1 | 0.864028069 | 0.021513131 |
| IMMP1L | 0.863825091 | 0.028646033 |
| NUDT22 | 0.863059155 | 0.022707665 |
| LOC650368 | 0.862256946 | 0.037558038 |
| CXorf40B | 0.861138956 | 0.01696905 |
| VRK3 | 0.861045286 | 0.029724691 |
| BM458889 | 0.860565533 | 0.005047771 |
| TRAPPC2 | 0.86004806 | 0.033697602 |
| EVI5L | 0.859767485 | 0.027671594 |
| TIMM8B | 0.859659658 | 0.013034466 |
| MAD1L1 | 0.859197382 | 0.04178199 |
| AUP1 | 0.859010684 | 0.033892809 |
| PSME3 | 0.858811339 | 0.021276239 |
| SYAP1 | 0.858789733 | 0.020211658 |
| C17orf37 | 0.858533968 | 0.005236734 |
| SDF4 | 0.858015445 | 0.046761042 |
| SEPHS1 | 0.85782498 | 0.045367877 |
| VCP | 0.857580272 | 0.03403151 |
| CUEDC2 | 0.857515774 | 0.011781918 |
| RAB1B | 0.857105915 | 0.021513131 |
| TESK1 | 0.856918034 | 0.034499794 |
| SCAND1 | 0.856762942 | 0.048042948 |
| PXMP4 | 0.856615528 | 0.023212086 |
| TAF10 | 0.856470704 | 0.013388843 |
| EDEM2 | 0.855935232 | 0.032099366 |
| FAM108A1 | 0.855247939 | 0.014621459 |
| ATG4A | 0.855244074 | 0.012848306 |
| ZHX2 | 0.854649443 | 0.017901484 |
| RPS19BP1 | 0.853933863 | 0.028517232 |
| ATP2C1 | 0.853236101 | 0.047977232 |
| BU163701 | 0.8529948 | 0.02304065 |
| SLC12A8 | 0.852040216 | 0.011093013 |
| YWHAB | 0.851833604 | 0.027671594 |
| ZDHHC16 | 0.851531892 | 0.022838314 |
| SLC25A20 | 0.850713854 | 0.026036175 |
| KGFLP1 | 0.850271545 | 0.008293669 |
| SETD8 | 0.849820706 | 0.001642406 |
| CCT8 | 0.84973626 | 0.037987629 |
| FAM108A1 | 0.849572082 | 0.007212646 |
| ANAPC1 | 0.849203701 | 0.005559513 |
| FAM108A1 | 0.849084535 | 0.018373974 |
| PXMP4 | 0.848583253 | 0.004939812 |
| BAD | 0.848161515 | 0.024431293 |
| ITGB5 | 0.847768169 | 0.026787682 |
| MNDA | 0.847664659 | 0.005236734 |
| GUK1 | 0.847177231 | 0.003376668 |
| VPS26B | 0.847138979 | 0.021978951 |
| C9orf95 | 0.846922406 | 0.031165234 |
| PRRG4 | 0.846917554 | 0.030341062 |
| DBT | 0.846707803 | 0.00177805 |
| KLHL8 | 0.846277306 | 0.015292876 |
| SPHK2 | 0.846145404 | 0.045446833 |
| C9orf91 | 0.845384168 | 0.049976388 |
| EXTL2 | 0.845276941 | 0.029114807 |
| DPM2 | 0.845012543 | 0.044672331 |
| THYN1 | 0.844892466 | 0.03193541 |
| UQCR10 | 0.844424123 | 0.011228688 |
| LRRC37A4 | 0.844281939 | 0.049528978 |
| DYNLL2 | 0.844023887 | 0.028428495 |
| DCAF6 | 0.843892496 | 0.031271057 |
| PIGM | 0.843470623 | 0.049272729 |
| GPS1 | 0.843266173 | 0.013981942 |
| MRPS15 | 0.843247448 | 0.005517031 |
| NDUFS8 | 0.84323736 | 0.030319965 |
| MVK | 0.843128265 | 0.045911014 |
| SLC27A5 | 0.843038069 | 0.042384422 |
| HES2 | 0.84292152 | 0.035171425 |
| ANO4 | 0.842849355 | 0.043817291 |
| APH1B | 0.842534932 | 0.049645855 |
| NR2F6 | 0.842330428 | 0.010790023 |
| HYLS1 | 0.842246013 | 0.01002127 |
| CLPTM1 | 0.84214004 | 0.020744124 |
| MPDU1 | 0.842131645 | 0.043536351 |
| ISOC2 | 0.841977557 | 0.023171788 |
| GGT3P | 0.841788224 | 0.013189186 |
| MPST | 0.841516679 | 0.033414951 |
| CNPY3 | 0.841414362 | 0.005108482 |
| KRIT1 | 0.841392292 | 0.00174522 |
| AURKA | 0.841307497 | 0.0305564 |
| NLRP12 | 0.841173035 | 0.013023004 |
| ASMTL | 0.841171611 | 0.004215848 |
| FKBP1B | 0.841055444 | 0.012075613 |
| GPX4 | 0.840650305 | 0.042626961 |
| METTL9 | 0.840608294 | 0.001384552 |
| STRADB | 0.840514947 | 0.00123277 |
| ICT1 | 0.840510115 | 0.01223402 |
| CACNG1 | 0.840062687 | 0.049177514 |
| CYB5R3 | 0.840056346 | 0.018077176 |
| FAM127C | 0.839659379 | 0.030268722 |
| GDF11 | 0.839538539 | 0.003082873 |
| MRPS11 | 0.839537601 | 0.019573988 |
| NSDHL | 0.83950299 | 0.017409331 |
| NOTCH2NL | 0.838811204 | 0.015771872 |
| DPP3 | 0.838568643 | 0.026214051 |
| KRT18 | 0.838509171 | 0.01388473 |
| RABEP2 | 0.838257342 | 0.000851853 |
| DNM1L | 0.83763876 | 0.040590068 |
| LRRC8A | 0.837546133 | 0.017409331 |
| HMBS | 0.837418825 | 0.03691428 |
| FAF1 | 0.836818117 | 0.030845471 |
| ADH5 | 0.836610297 | 0.028875768 |
| UBE2G1 | 0.836142848 | 0.02304065 |
| PI4KA | 0.835891111 | 0.015405623 |
| PDXP | 0.835051784 | 0.036359887 |
| TUBA1B | 0.834576581 | 0.041389396 |
| MFSD5 | 0.833636665 | 0.031732952 |
| RAD51C | 0.833577732 | 0.01854546 |
| GNAQ | 0.83356103 | 0.038320188 |
| MED27 | 0.833230241 | 0.047416177 |
| PARK7 | 0.833221229 | 0.021595997 |
| ORAI3 | 0.83275417 | 0.008748175 |
| TYMS | 0.832657104 | 0.024222516 |
| APEH | 0.832486551 | 0.006885993 |
| SEC31A | 0.832399374 | 0.013774066 |
| CTTN | 0.831850174 | 0.02304065 |
| AGBL5 | 0.8318386 | 0.015720233 |
| CTBS | 0.831834001 | 0.032014308 |
| FAM108A1 | 0.831541537 | 0.005215863 |
| CDCA5 | 0.831038094 | 0.029693044 |
| DHRS7B | 0.830702782 | 0.024222516 |
| TALDO1 | 0.830676889 | 0.02652771 |
| C11orf73 | 0.82997419 | 0.019294801 |
| FAM86A | 0.829938985 | 0.04201855 |
| CHMP2A | 0.82958152 | 0.021276239 |
| EIF1AX | 0.829503967 | 0.047540178 |
| RPL10 | 0.829315857 | 0.035375086 |
| CLPP | 0.829181475 | 0.006167831 |
| SYVN1 | 0.829111508 | 0.03881077 |
| SNTB2 | 0.829048699 | 0.036141617 |
| CKS2 | 0.828513233 | 0.020302022 |
| XPNPEP1 | 0.827870783 | 0.048038477 |
| DOLK | 0.827792425 | 0.024431293 |
| PIN1 | 0.826987271 | 0.042873015 |
| ALG3 | 0.826801645 | 0.019642805 |
| VPS28 | 0.826689738 | 0.038450531 |
| SEPW1 | 0.826488075 | 0.028921216 |
| ECT2 | 0.826312519 | 0.017001773 |
| TADA3 | 0.826146388 | 0.005360918 |
| DAP | 0.826052102 | 0.02453605 |
| ESRRG | 0.826050431 | 0.038104931 |
| TMED1 | 0.825927961 | 0.011810705 |
| ATL1 | 0.825600842 | 0.004376336 |
| CCT7 | 0.824821897 | 0.035093179 |
| USP33 | 0.824382651 | 0.037094207 |
| ARF1 | 0.824102263 | 0.000913969 |
| MRPL21 | 0.82399425 | 0.042235076 |
| LLPH | 0.823842495 | 0.041718103 |
| SURF4 | 0.823808768 | 0.037858241 |
| PSMB7 | 0.823657427 | 0.008741365 |
| RNF181 | 0.823516424 | 0.041243707 |
| RUVBL2 | 0.823105141 | 0.006082946 |
| ZNF813 | 0.8225834 | 0.002485155 |
| SMPD1 | 0.822285627 | 0.016109021 |
| RDH5 | 0.821440876 | 0.041148152 |
| ZNF577 | 0.821342363 | 0.013023004 |
| C10orf47 | 0.820630351 | 0.025932276 |
| CTNNBIP1 | 0.820206052 | 0.022670328 |
| EPB41L4B | 0.820033352 | 0.04178199 |
| TIPRL | 0.819751811 | 0.026787682 |
| TPD52 | 0.819576511 | 0.007700834 |
| PMEPA1 | 0.819212861 | 0.001465497 |
| MED27 | 0.818811201 | 0.014695514 |
| SLC43A1 | 0.818493587 | 0.020557243 |
| CHRNA2 | 0.818420262 | 0.00527852 |
| FAM129B | 0.818028506 | 0.033221642 |
| NOL3 | 0.817931957 | 0.047044873 |
| SUOX | 0.817774877 | 0.021912298 |
| PSMC4 | 0.81721706 | 0.038701981 |
| AKR1A1 | 0.816923455 | 0.034269328 |
| MAZ | 0.816831667 | 0.011900729 |
| AK125546 | 0.816765022 | 0.018643898 |
| TTC12 | 0.816545766 | 0.036050865 |
| CD151 | 0.816470238 | 0.035093179 |
| LIMS1 | 0.816385226 | 0.036035288 |
| ACOT8 | 0.816172093 | 0.006197525 |
| WDR23 | 0.815551005 | 0.017409331 |
| PHKB | 0.814852955 | 0.049749378 |
| POLD4 | 0.814660472 | 0.040267392 |
| ASMTL | 0.814136671 | 0.003284875 |
| LCLAT1 | 0.814082663 | 0.028406725 |
| CYTH1 | 0.814063862 | 0.037292531 |
| ASMTL | 0.814029674 | 0.020573112 |
| CHCHD5 | 0.813928609 | 0.012528213 |
| ST6GAL1 | 0.81387138 | 0.033973641 |
| SETD8 | 0.813708412 | 0.011228688 |
| GLB1L3 | 0.813146835 | 0.007700834 |
| ELMO2 | 0.812598129 | 0.003183961 |
| PLXNB2 | 0.812581339 | 0.022068522 |
| AP2S1 | 0.811931681 | 0.031188009 |
| FAM108A1 | 0.811683764 | 0.027418646 |
| YBEY | 0.811426832 | 0.048393655 |
| MAR03 | 0.811300933 | 0.000320196 |
| GGT8P | 0.811136748 | 0.046912127 |
| SLC2A4RG | 0.810380186 | 0.000851853 |
| MTFP1 | 0.810230085 | 0.003082873 |
| AI557257 | 0.809708489 | 0.008653163 |
| SLC25A10 | 0.809665784 | 0.03282713 |
| TUBG1 | 0.809549795 | 0.017473257 |
| NUSAP1 | 0.809172528 | 0.043760398 |
| TPM4 | 0.808965496 | 0.026214051 |
| ALKBH7 | 0.808955002 | 0.021912298 |
| COX5B | 0.808818476 | 0.045959677 |
| GOT1 | 0.808390687 | 0.000501384 |
| ATL1 | 0.808215593 | 0.014819436 |
| REPS2 | 0.807921886 | 0.004933344 |
| DNAJC10 | 0.807823423 | 0.043647293 |
| TMEM187 | 0.807255496 | 0.03335651 |
| DAG1 | 0.806625108 | 0.005789922 |
| TMEM192 | 0.805790563 | 0.006968762 |
| PPP2R2D | 0.805739033 | 0.028687665 |
| ZNF697 | 0.8048731 | 0.029186693 |
| PDCD4 | 0.804849687 | 0.00401477 |
| SCAND1 | 0.804791375 | 0.012112494 |
| PPDPF | 0.804630469 | 0.041404117 |
| ARHGAP28 | 0.80460325 | 0.037265804 |
| SELS | 0.804418095 | 0.04346773 |
| KRT18 | 0.803965873 | 0.000851853 |
| ZNF286A | 0.803786991 | 0.036008076 |
| ADI1 | 0.803773954 | 0.034499794 |
| ANAPC11 | 0.803729778 | 0.012519677 |
| MRPS18A | 0.803704827 | 0.013542121 |
| LAMTOR2 | 0.803109038 | 0.012516033 |
| FAM195A | 0.802979453 | 0.025656578 |
| GLB1L3 | 0.80215765 | 0.016664522 |
| CRK | 0.80181376 | 0.044941333 |
| MYO1C | 0.801802332 | 0.01022877 |
| SLC35B1 | 0.801541585 | 0.016664522 |
| TMEM187 | 0.80151538 | 0.015898287 |
| KCNH6 | 0.801307274 | 0.031772402 |
| PPP1R7 | 0.801266108 | 0.009065591 |
| TBC1D16 | 0.801240956 | 0.029657451 |
| AKR1A1 | 0.800750318 | 0.035509026 |
| STX8 | 0.800321317 | 0.02377511 |
| TUBG1 | 0.799990069 | 0.022235123 |
| CDC42EP2 | 0.799379912 | 0.008748175 |
| DNAJC3 | 0.798796359 | 0.006977935 |
| TMED9 | 0.798712532 | 0.022713764 |
| YIF1B | 0.798641134 | 0.001243112 |
| OAZ3 | 0.798275061 | 0.012334708 |
| ARHGAP28 | 0.798148865 | 0.043434647 |
| SPCS2 | 0.797845624 | 0.028796053 |
| GGT3P | 0.797571097 | 0.021339554 |
| INO80B-WBP1 | 0.797537726 | 0.009123792 |
| PAOX | 0.797507419 | 0.021172122 |
| CALR | 0.7974352 | 0.000714052 |
| SLC2A10 | 0.797325426 | 0.015286608 |
| EXOSC1 | 0.797205609 | 0.00527852 |
| PDPK1 | 0.797163884 | 0.040210137 |
| XAB2 | 0.797050857 | 0.039224769 |
| UBE2C | 0.796913234 | 0.010995831 |
| REPS2 | 0.796666877 | 0.002375176 |
| APLP2 | 0.796662406 | 0.027256315 |
| C10orf75 | 0.796402754 | 0.022262207 |
| MRPL39 | 0.795225584 | 0.03647484 |
| TNFAIP8 | 0.795178929 | 0.01673378 |
| GGT1 | 0.795169055 | 0.03161553 |
| PPM1K | 0.795166434 | 0.022020298 |
| TEX264 | 0.794879759 | 0.023435722 |
| DHTKD1 | 0.79475456 | 0.042172051 |
| DNM1L | 0.79433104 | 0.009657327 |
| C2orf79 | 0.79426441 | 0.008391043 |
| PIG8 | 0.794089355 | 0.016577735 |
| DEXI | 0.794086308 | 0.011565795 |
| PLEKHB2 | 0.793742674 | 0.014819436 |
| KLK15 | 0.793581613 | 0.015898287 |
| ASTN2 | 0.793262098 | 0.012197311 |
| RNF103 | 0.792767985 | 0.047540178 |
| MANF | 0.79180283 | 0.036359887 |
| LOC550112 | 0.791510333 | 0.00104475 |
| BRI3BP | 0.791401792 | 0.003698779 |
| C22orf13 | 0.791003347 | 0.006464976 |
| TCP1 | 0.790488708 | 0.043434647 |
| YIF1B | 0.789796573 | 0.003376668 |
| LCLAT1 | 0.789604437 | 0.022913428 |
| KLK15 | 0.789265156 | 0.030704479 |
| C1orf116 | 0.789141015 | 0.007165272 |
| PEMT | 0.78905188 | 0.011081309 |
| SLC25A42 | 0.788579717 | 0.030356045 |
| ERBB3 | 0.788344591 | 0.020744124 |
| ATL1 | 0.787329582 | 0.005059606 |
| FBXL8 | 0.787043452 | 0.004116571 |
| STARD7 | 0.78703105 | 0.043434647 |
| CASP6 | 0.787024749 | 0.002247005 |
| TMEM134 | 0.786656243 | 0.04595859 |
| PRKCH | 0.786623092 | 0.002375176 |
| MRPS23 | 0.784912878 | 0.02412336 |
| GPX4 | 0.784841595 | 0.009631183 |
| CHMP2A | 0.784781735 | 0.02396778 |
| PSMD8 | 0.784191893 | 0.000251821 |
| ZNF264 | 0.783077176 | 0.011421095 |
| CMAS | 0.782525765 | 0.019794807 |
| ACAA1 | 0.782027185 | 0.030828237 |
| PPIA | 0.781536707 | 0.039420972 |
| CPT2 | 0.781310809 | 0.01146988 |
| PDLIM5 | 0.781139394 | 0.021276239 |
| EHHADH | 0.781079067 | 0.005526016 |
| FKBP4 | 0.780932067 | 0.031732952 |
| COX6B1 | 0.780858818 | 0.007700834 |
| G3BP2 | 0.780184075 | 0.037704265 |
| COX8A | 0.779058536 | 0.03576109 |
| C20orf108 | 0.778827505 | 0.040631981 |
| PCYOX1 | 0.778552077 | 0.011081309 |
| TRIM5 | 0.777015045 | 0.009594522 |
| SMPDL3B | 0.776886682 | 0.034782073 |
| LOC81691 | 0.776522021 | 0.048223369 |
| C6orf129 | 0.776331185 | 0.012374578 |
| MPST | 0.775664842 | 0.002050752 |
| TCEB2 | 0.775314338 | 0.007165272 |
| AP3M1 | 0.775300253 | 0.020719803 |
| EIF3CL | 0.775198505 | 0.033973641 |
| H3F3A | 0.77517812 | 0.027418646 |
| MIF | 0.775173794 | 0.02760136 |
| AP3M1 | 0.774791665 | 0.020485671 |
| SLC35E1 | 0.773972263 | 0.009451092 |
| DUS1L | 0.773743853 | 0.01157044 |
| DOPEY2 | 0.77361088 | 0.01814117 |
| TRAPPC5 | 0.773057967 | 0.003698779 |
| HMOX2 | 0.772442453 | 0.00015085 |
| RNF170 | 0.771958673 | 0.021416515 |
| YME1L1 | 0.771844519 | 0.021488151 |
| NOMO3 | 0.770892709 | 0.003698779 |
| TADA3 | 0.76995594 | 0.008944001 |
| UBE2J1 | 0.769738536 | 0.038868178 |
| CMAS | 0.769601443 | 0.03881077 |
| SEC61B | 0.769383263 | 0.043434647 |
| CAMK1 | 0.769302312 | 0.018538843 |
| CD151 | 0.769044367 | 0.017901484 |
| PGM1 | 0.768316759 | 0.046334928 |
| GPR56 | 0.768237084 | 0.027184609 |
| PPP1CA | 0.767959345 | 0.034268582 |
| CACYBP | 0.767234869 | 0.041461599 |
| TMEM87B | 0.766446856 | 0.004933344 |
| LCLAT1 | 0.766247461 | 0.011017252 |
| ALDH4A1 | 0.765893006 | 0.016393538 |
| CENPM | 0.765578545 | 0.016664522 |
| GPR56 | 0.765525746 | 0.0305564 |
| PPIA | 0.765525288 | 0.039544014 |
| YIPF1 | 0.764462908 | 0.014695514 |
| TMBIM1 | 0.764138125 | 0.049443971 |
| P53TG1 | 0.763752549 | 0.009315835 |
| SEC14L5 | 0.763705337 | 0.002050752 |
| OCEL1 | 0.763699725 | 0.002545264 |
| FEV | 0.76223326 | 0.023479138 |
| KRT18 | 0.762157138 | 0.003698779 |
| ACSL3 | 0.761334343 | 0.006160087 |
| GMPPB | 0.761329034 | 0.006376529 |
| WIPI1 | 0.760943597 | 0.011810705 |
| SNRPD3 | 0.760876218 | 0.048300568 |
| ABHD11 | 0.759722066 | 0.004053194 |
| ENDOG | 0.759675015 | 0.0049649 |
| GMPPB | 0.759367415 | 0.042384422 |
| TM9SF1 | 0.759109323 | 0.021276239 |
| FMO5 | 0.758880962 | 0.042867004 |
| C6orf125 | 0.758877225 | 0.001079261 |
| STT3A | 0.758214129 | 0.016664522 |
| HMG20B | 0.758111139 | 0.023852908 |
| COPE | 0.757850933 | 0.003698779 |
| PRADC1 | 0.756676324 | 5.95E-05 |
| TUBB8 | 0.756128783 | 0.018072947 |
| DISC1 | 0.755693337 | 0.010435124 |
| RBX1 | 0.755479688 | 0.010995831 |
| PTTG1IP | 0.755368408 | 0.00401477 |
| MTOR | 0.755363896 | 0.001886133 |
| LOC388588 | 0.755228918 | 0.013034466 |
| TM9SF1 | 0.754226057 | 0.03385751 |
| FKBP2 | 0.754198011 | 0.005520814 |
| RNF181 | 0.75352064 | 0.001812821 |
| HYOU1 | 0.753128707 | 0.004072047 |
| BTD | 0.752418567 | 0.006197525 |
| NOMO1 | 0.751606156 | 0.024222516 |
| PCCB | 0.75113973 | 0.011006194 |
| TMEM41A | 0.750836385 | 0.011511408 |
| PAQR4 | 0.750384023 | 0.045424831 |
| IVD | 0.750121213 | 0.021276239 |
| MTMR2 | 0.749785046 | 0.000251821 |
| C10orf32-AS3MT | 0.748609443 | 0.025372964 |
| TM9SF1 | 0.748517236 | 0.003941132 |
| POLR2J2 | 0.74842672 | 0.039420972 |
| CHP | 0.747459784 | 0.003409417 |
| SLC25A20 | 0.747352923 | 0.002050752 |
| PDLIM5 | 0.746454032 | 0.028490736 |
| CKS2 | 0.745910283 | 0.018071717 |
| CAMKK2 | 0.745885024 | 0.008819737 |
| SSR4 | 0.745248356 | 0.011081309 |
| COPE | 0.745079493 | 0.000653982 |
| CKMT1B | 0.745056083 | 0.006035109 |
| GGT1 | 0.745044408 | 0.040062438 |
| PCTP | 0.743934203 | 0.001408173 |
| TXNDC17 | 0.743705734 | 0.008334469 |
| PSMA6 | 0.743505064 | 0.031772402 |
| FXYD3 | 0.74347795 | 0.016197406 |
| DKFZp761K0816 | 0.742427779 | 0.04178199 |
| EAF2 | 0.742132685 | 0.044470188 |
| RHOBTB3 | 0.741353067 | 0.013190584 |
| ECI2 | 0.74124219 | 0.039122195 |
| CCNB2 | 0.740868626 | 0.00496537 |
| DTYMK | 0.740301511 | 0.011081309 |
| C8orf55 | 0.740290477 | 0.01966387 |
| P4HB | 0.740128603 | 0.003281799 |
| PMM1 | 0.740037357 | 0.003994954 |
| CORO2A | 0.739032184 | 0.007435903 |
| SLC15A2 | 0.738985604 | 0.042463694 |
| FXYD3 | 0.737870416 | 0.020668981 |
| SLC1A5 | 0.737853152 | 0.000251821 |
| NDUFA12 | 0.737514202 | 0.038795359 |
| HSPA5 | 0.736984223 | 0.027038219 |
| C1orf122 | 0.735290651 | 0.00177805 |
| TSPO | 0.735240691 | 0.01674289 |
| C9orf140 | 0.735037052 | 0.00054927 |
| SYTL1 | 0.734200637 | 0.029907092 |
| ZNF761 | 0.733229874 | 0.005052699 |
| ZNF761 | 0.731208672 | 0.005421536 |
| UBE2C | 0.729522394 | 0.014456994 |
| BCAT2 | 0.729417615 | 0.006835141 |
| TPT1 | 0.729274086 | 0.048300568 |
| ZNF217 | 0.728960863 | 0.026214051 |
| ALDH7A1 | 0.728144228 | 0.020030315 |
| PLEKHB2 | 0.727310266 | 0.010435124 |
| REPS2 | 0.727199934 | 0.001921816 |
| THYN1 | 0.726162449 | 0.001417996 |
| SHROOM3 | 0.724816731 | 0.006309475 |
| BTBD11 | 0.724725926 | 0.021295843 |
| TSPO | 0.724670842 | 0.023171788 |
| C17orf28 | 0.723615318 | 0.003941132 |
| Tmp21-II | 0.722623753 | 0.037116407 |
| ECI1 | 0.72150209 | 0.000906926 |
| UAP1 | 0.720972998 | 0.020629213 |
| TMEM8A | 0.720952859 | 0.002385625 |
| NSF | 0.72060628 | 0.013695799 |
| ZNF350 | 0.720301688 | 0.021232297 |
| SLCO2A1 | 0.719838844 | 0.021079552 |
| MEX3B | 0.71937743 | 0.000320196 |
| THYN1 | 0.719271417 | 0.00527852 |
| ABHD11 | 0.719124137 | 0.014057792 |
| ALDH4A1 | 0.719026198 | 0.017409331 |
| PTPLAD1 | 0.718370216 | 0.031840874 |
| TOP2A | 0.718228934 | 0.027544421 |
| CR613620 | 0.717670393 | 0.022371068 |
| UBB | 0.717554013 | 0.031613129 |
| TPD52 | 0.716545194 | 0.028428495 |
| C19orf10 | 0.71593434 | 0.021978951 |
| SERINC2 | 0.714863213 | 0.004241216 |
| PXDN | 0.713498595 | 0.002672078 |
| ALDH4A1 | 0.713465165 | 0.009433357 |
| CR613620 | 0.712710234 | 0.01696905 |
| SNRPN | 0.711628044 | 0.026214051 |
| CDK6 | 0.71081847 | 0.009164983 |
| ABAT | 0.710427858 | 0.026836192 |
| TRIM68 | 0.709205507 | 0.00565934 |
| ZDHHC8P | 0.708521576 | 0.016681399 |
| ECI2 | 0.7080007 | 0.03193541 |
| TM9SF2 | 0.707694336 | 0.005872553 |
| COX6A1 | 0.707310642 | 0.002837138 |
| GOLM1 | 0.707093056 | 0.02183485 |
| PDIA6 | 0.706891082 | 0.028050653 |
| HIST1H4J | 0.706506392 | 0.03576109 |
| IDI1 | 0.705818796 | 0.02487282 |
| SPCS2 | 0.705655037 | 0.013774066 |
| RPN2 | 0.704394321 | 0.026597649 |
| CD320 | 0.703171528 | 0.008145917 |
| TMEM141 | 0.702766685 | 0.007700834 |
| PMEPA1 | 0.702595185 | 0.000984446 |
| ATG4B | 0.701616007 | 0.005790025 |
| PPAPDC2 | 0.701094411 | 0.004376336 |
| ZNF511 | 0.700554075 | 5.21E-06 |
| KRT8 | 0.700233977 | 0.002247005 |
| RAB27A | 0.700050379 | 0.0305564 |
| RNF157 | 0.699024744 | 0.048031398 |
| KRT18 | 0.697723433 | 0.006396208 |
| REPS2 | 0.697644281 | 0.035187956 |
| PAOX | 0.693987037 | 0.008145917 |
| TFPT | 0.693767661 | 0.001129515 |
| PDIA4 | 0.693725516 | 0.003376668 |
| ATP5C1 | 0.693285555 | 0.039544014 |
| GINS2 | 0.692989284 | 0.000785745 |
| SPCS2 | 0.691460448 | 0.045657296 |
| DHCR7 | 0.691332898 | 0.015373181 |
| NAAA | 0.691204354 | 0.00174522 |
| MTFP1 | 0.690953484 | 0.004029155 |
| KIAA0319L | 0.689744785 | 0.0068644 |
| HM13 | 0.688942399 | 0.009349945 |
| SLC35F2 | 0.688736493 | 0.003423887 |
| LPAR3 | 0.688632265 | 0.033251175 |
| DBNDD1 | 0.688531007 | 0.021935501 |
| DEGS1 | 0.686304221 | 0.022262207 |
| PTPRM | 0.686237565 | 0.031100708 |
| RPL10 | 0.685563691 | 0.040062438 |
| CNPY3 | 0.68506055 | 0.003446259 |
| AQP11 | 0.683731897 | 0.042384422 |
| DHCR7 | 0.683175478 | 0.005695339 |
| IMPA2 | 0.680997429 | 0.000621825 |
| KRT8 | 0.680693156 | 0.000896583 |
| EEF1B2 | 0.678920022 | 0.033150145 |
| FKBP5 | 0.678196332 | 0.041593402 |
| STRA13 | 0.677787472 | 0.000173861 |
| C9orf152 | 0.677243407 | 0.042384422 |
| ERGIC1 | 0.67723853 | 0.00176388 |
| ST6GALNAC1 | 0.67611172 | 0.000637198 |
| SPDEF | 0.676007971 | 0.037481755 |
| CD151 | 0.675715513 | 0.017010138 |
| SHROOM3 | 0.675471427 | 0.000174766 |
| REPS2 | 0.674656868 | 0.008909749 |
| ERP29 | 0.674570618 | 0.019652703 |
| PNKP | 0.673337929 | 0.001727383 |
| REPS2 | 0.672004271 | 0.004014227 |
| ADM2 | 0.670254623 | 0.001304032 |
| YIPF1 | 0.667361065 | 0.000504031 |
| VGLL3 | 0.666034513 | 0.048294954 |
| CYB561 | 0.664931596 | 0.007700834 |
| PRSS8 | 0.663277807 | 0.013695799 |
| KRT8 | 0.662580642 | 0.002078917 |
| TMED10 | 0.662527695 | 0.021295843 |
| C1orf116 | 0.66232897 | 0.042218244 |
| CKMT1B | 0.662065342 | 0.001172054 |
| KRT8 | 0.660792121 | 0.005681868 |
| C9orf140 | 0.660440324 | 0.000173861 |
| PTPRN2 | 0.656335775 | 6.65E-05 |
| GGTLC2 | 0.656163096 | 0.017901484 |
| C10orf58 | 0.656021361 | 0.002141938 |
| SLC26A3 | 0.654619955 | 0.037704265 |
| KRT18 | 0.653079075 | 0.029181768 |
| GNE | 0.652624528 | 0.003941132 |
| TPT1 | 0.651646556 | 0.028428495 |
| ZDHHC8P | 0.649869857 | 0.04178199 |
| APOF | 0.649566309 | 0.004008349 |
| CKMT1B | 0.648275061 | 0.00585774 |
| PDIA3P | 0.646638181 | 0.012638905 |
| ALDH1A3 | 0.646278188 | 0.016664522 |
| GDF11 | 0.644315682 | 0.02860102 |
| TSPAN1 | 0.643118023 | 0.014819436 |
| FLJ40504 | 0.64211759 | 0.002277593 |
| ZDHHC8P | 0.641911754 | 0.023852908 |
| ACADL | 0.640435494 | 0.033697602 |
| CORO1B | 0.637925861 | 5.95E-05 |
| ALOX15B | 0.637357318 | 0.034547055 |
| CYB561 | 0.631546862 | 0.00202832 |
| TGM3 | 0.630295034 | 0.0331473 |
| FAM174B | 0.630197548 | 0.001823287 |
| NPR3 | 0.630099192 | 0.037895023 |
| LOC729860 | 0.627670145 | 0.001085462 |
| CYB5A | 0.623140496 | 0.019642805 |
| AP2S1 | 0.620681004 | 0.000251821 |
| TMEM125 | 0.620507001 | 0.001449121 |
| DCXR | 0.619791375 | 0.001243112 |
| NANS | 0.619723741 | 0.004933344 |
| GJB1 | 0.61695409 | 0.003719722 |
| AGTRAP | 0.616674597 | 4.46E-05 |
| LOC728606 | 0.616522947 | 0.005935095 |
| AGTRAP | 0.616114001 | 0.000185535 |
| SEC14L2 | 0.608966326 | 0.000726808 |
| C1orf116 | 0.601508891 | 0.014609159 |
| SORD | 0.599758519 | 0.01100682 |
| FASN | 0.597106288 | 0.014057792 |
| CORO1B | 0.594527718 | 3.04E-06 |
| FABP5 | 0.592945503 | 0.035591913 |
| SLC35F2 | 0.591802737 | 0.000276818 |
| MT1JP | 0.587490582 | 0.007700834 |
| PPM1H | 0.585283058 | 0.00177805 |
| ALDH4A1 | 0.585157685 | 0.000478898 |
| KLK4 | 0.582170723 | 0.045629272 |
| MT1IP | 0.582158659 | 0.028428495 |
| CBS | 0.581939546 | 0.019652703 |
| AFF3 | 0.57776287 | 0.019450232 |
| RAB27A | 0.576728515 | 0.02453605 |
| HBB | 0.576722717 | 0.045207965 |
| FAM189A2 | 0.575694092 | 0.000173861 |
| DCN | 0.573535651 | 0.030107918 |
| ENDOD1 | 0.570680729 | 0.000896583 |
| PMEPA1 | 0.568151459 | 0.00177805 |
| SORD | 0.565828364 | 0.031772402 |
| PNKD | 0.565419806 | 0.00177805 |
| NAAA | 0.563176572 | 0.001076972 |
| PMEPA1 | 0.561805594 | 0.000851853 |
| PAK1IP1 | 0.557387705 | 0.031217415 |
| SORD | 0.556475281 | 0.014550435 |
| NAAA | 0.554666478 | 0.000913969 |
| PCA3 | 0.554280159 | 0.017124452 |
| MS4A8B | 0.551132858 | 0.001911384 |
| SLC2A12 | 0.549577816 | 0.000955745 |
| ABCC4 | 0.548333024 | 0.016416875 |
| TMPRSS2 | 0.547754955 | 0.040050887 |
| ACAD8 | 0.535405204 | 0.000260145 |
| MT1E | 0.532050236 | 0.021139635 |
| TRPM4 | 0.525520077 | 0.001605567 |
| CECR6 | 0.519464475 | 0.00046444 |
| MT1H | 0.517005185 | 0.025232597 |
| EBP | 0.513724061 | 0.000820377 |
| PAGE4 | 0.511161197 | 0.025762599 |
| DHCR24 | 0.504035041 | 0.000266087 |
| MBOAT2 | 0.503732878 | 8.34E-06 |
| RAB3B | 0.501778679 | 0.000369376 |
| SMS | 0.493837786 | 0.002141938 |
| GLB1L2 | 0.493260638 | 7.91E-06 |
| SPON2 | 0.493026614 | 0.039420972 |
| SCD | 0.492641824 | 0.013968272 |
| LRRC26 | 0.488625783 | 0.00035354 |
| MT1F | 0.484183235 | 0.004008349 |
| SMS | 0.47950789 | 0.000851853 |
| LRRC26 | 0.4787196 | 0.000251821 |
| ABCC4 | 0.474038544 | 0.003608711 |
| DBI | 0.47344772 | 0.00017068 |
| SMS | 0.466526486 | 0.000896583 |
| LRRC26 | 0.460558399 | 0.000188542 |
| AMACR | 0.459272876 | 0.000185535 |
| SMS | 0.453173258 | 0.000363858 |
| TMSB15B | 0.451462839 | 5.95E-05 |
| SMS | 0.449814906 | 0.000173861 |
| ALOX15B | 0.441907503 | 0.039398483 |
| ORM2 | 0.441622501 | 0.027685512 |
| MBOAT2 | 0.441534709 | 4.11E-06 |
| AMACR | 0.428363621 | 0.001170749 |
| KLK3 | 0.422053884 | 0.027847159 |
| GNMT | 0.421645464 | 0.006464976 |
| DBI | 0.419408075 | 0.000173222 |
| AMACR | 0.41509243 | 0.000548699 |
| MT1G | 0.407523035 | 0.008291519 |
| TMSB15B | 0.404812213 | 5.95E-05 |
| PHGR1 | 0.395393204 | 3.77E-07 |
| ANPEP | 0.388815415 | 0.03193541 |
| MYBPC1 | 0.382091097 | 0.000152368 |
| GDF15 | 0.36166001 | 0.003166353 |
| MYBPC1 | 0.359761494 | 0.001609792 |
| TMSB15A | 0.337598714 | 5.98E-05 |
| NCAPD3 | 0.226768024 | 8.91E-07 |

**Supplementary Table 4 – Table of validated androgen-regulated genes with details of androgen receptor binding sites from chromatin immunoprecipitation sequencing studies in VCaP or LNCaP cell lines and human castration-resistant prostate cancer samples**

| Gene | ARBS in cell lines [5] | ARBS in CRPC clinical samples [6] | Androgen regulated in cell lines [5,7] | Androgen regulated in xenografts [6] | Function |
| --- | --- | --- | --- | --- | --- |
| *AMACR* | V and L | 2 of 5 patients | Yes | Yes | Fatty acid metabolism |
| *FAM129A* | V and L | 2 of 5 patients | No | No | Suppressing apoptosis |
| *KIAA0101* | V | 0 | Yes | Yes | DNA damage repair |
| *RAB27A* | V and L | 2 of 5 patients | Yes | No | Tumour microenvironment |

ARBS = androgen receptor binding site; CRPC = castration-resistant prostate cancer; v = VCaP; L = LNCaP
