# Supplementary material for: The Early Effects of Rapid Androgen Deprivation on Human Prostate Cancer
Source: Eur Urol. 2016 Aug;70(2):214–8. doi: 10.1016/j.eururo.2015.10.042 (PMC4926724; doi:10.1016/j.eururo.2015.10.042)

**Supplementary Fig. 1 – Degarelix lowers serum, urine, and intraprostatic androgens. (a) Study flowchart: 15 patients with messenger RNA of sufficient quality were compared by gene expression profiling with 20 untreated matched controls (Supplementary Table 1). (b) Serum testosterone before and 7 d after medical castration with degarelix (*p* < 0.0001; paired *t*-test; *n* = 27). (c) Intraprostatic testosterone concentration in prostate harvested 7 d after degarelix treatment and untreated matched control samples (*p* = 0.0002; Mann-Whitney test; *n* = 10). GC = gas chromatography; LC = liquid chromatography; mRNA = messenger RNA; MS = mass spectrometry; s.c. = subcutaneously.**


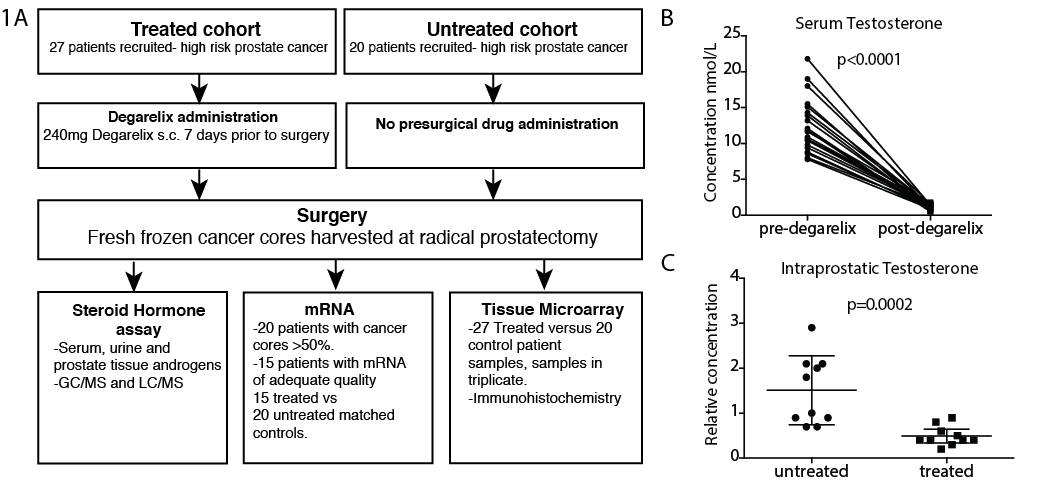

Supplement: Supplementary file 3 [file mmc3.doc]
